# Characteristics of Kevlar and Glass Fibers, the Effects of Physical and Methodological Parameters, and the Influence of Hybridization with Vegetable Fibers on Impact Properties of Composites—A Review

**DOI:** 10.3390/polym18151837

**Published:** 2026-07-27

**Authors:** Marilena Manea, Anton Hadăr, Camelia Cerbu

**Affiliations:** 1 Faculty of Industrial Engineering and Robotics, National University of Science and Technology Politehnica Bucharest, B-dul Splaiul Independenţei No. 313, 060042 Bucharest, Romania; marilena.manea31@gmail.com; 2 National Institute for Aerospace Research ‘Elie Carafoli’, B-dul Iuliu Maniu No. 220, 061126 Bucharest, Romania; 3 Technical Sciences Academy of Romania, B-dul Dacia No. 26, 030167 Bucharest, Romania; 4 Academy of Romanian Scientists, Ilfov Street No. 3, 050045 Bucharest, Romania; 5 Faculty of Mechanical Engineering, Transilvania University of Brașov, B-dul Eroilor No. 29, 500036 Brasov, Romania

**Keywords:** Kevlar, glass fiber, vegetable fibers, nose-shape geometry, temperature, UV radiation, impact, manufacturing parameters, moisture

## Abstract

Integration of composites into the fabrication process of structural assemblies within the aerospace, automotive, marine or civil engineering industries represents a rational solution adopted by leading companies which are guided by the necessity for novel low-weight, high-strength, and high-stiffness materials. During the manufacturing process and throughout the service life, fiber-reinforced polymer structures are subjected to impact loading, either accidentally or as an inherent requirement of the operational cycle. Firstly, general aspects regarding impact loading and some parameters used for its characterization are briefly described. Recent progress regarding the influence of the stacking sequence, fiber type, and impactor geometry on the impact performance of Kevlar and glass fiber reinforced composite materials is emphasized. Additionally, the effects of environmental factors (such as temperature, UV radiation, or humidity) on the impact energy absorbed by polymers reinforced with each of the two types of synthetic fibers are presented. Finally, the importance of directing the researcher’s judgment towards improving the characteristics of materials subjected to impact, from a sustainable perspective, is motivated through the presentation of the impact behavior of polymer composites reinforced with Kevlar fibers or glass fibers hybridized with vegetable fibers.

## 1. Introduction

To design innovative materials aimed at achieving the required performance of engineering structures, there have been numerous approaches over time. In this regard, the concept of composite materials was introduced to encompass different types of materials used to create and optimize industrial technologies. A composite material is a structural system that consists of multiple macroscopically identifiable constituents that operate together to achieve improved mechanical performance [1]. The rationale for adopting novel materials and customized production methods can be expressed in terms of high strength, improved stiffness, and the shortage of tailored materials for different types of applications.

Like every complex engineering achievement, composite materials have advantages and disadvantages. Manufacturing of complex shapes, the combination of different constituent materials, and the simplification of the production processes are among the numerous advantages. Also, strength, stiffness, thermal behavior, and electrical properties can be designed. The limitations include: high material costs; recycling processes that are not well developed; sensitivity of their properties to temperature changes, moisture absorption, fire, lightning strikes, and UV radiation [1].

Composite materials are extensively used in the fabrication processes of aircraft and spacecraft structural assemblies, automotive, marine, and civil engineering industries [2,3]. During the service life or the manufacturing stage, a fiber-reinforced structure is subjected to accidental impact damage. Thus, potential sources of impact are stones, dust, or small pieces of debris lofted by aircraft tires during take-off or landing operations on unpaved runways [4]. A relevant technical publication [5] refers to the effects of these types of objects as Foreign Object Damage (FOD). The primary concern regarding the capacity of the composite material structure to withstand impact loads is the detection of the damage resulting from such dynamic loading. The severity of the defect is not always identifiable by visual inspection of the impacted surface. Although metals show visible deformations, some laminates bounce back close to their initial geometry even if there is damage, such as cracks and delamination, which are located between the inner laminas of the material [1]. Depending on the type of inspection required and the tools used for non-destructive examination, impact loading generates four types of damage: special defects, barely visible impact damage (BVID), minor visible impact damage (MVID), and Large Visible Impact Damage (LVID) [6].

Although fiber-reinforced composite materials exhibit outstanding performance under tensile, compressive, or bending loading conditions, their capability to withstand impact loads is lower compared to metals because of their brittleness [7]. Composite laminates reinforced with glass, carbon, or aramid fibers are representative in terms of strength and stiffness performance coupled with low weight, making them suitable for impact applications. There are various factors that influence the impact behavior of composite laminates, including projectile shape and mass, angular orientation of the lamina fibers, matrix toughness, and stacking sequence [8]. Also, environmental factors control the out-of-plane impact strength because long-term exposure to hygrothermal conditions affects the matrix physicochemical state in composite laminates [9].

Three common types of reinforcements used to investigate the low-velocity impact response, including damage mechanisms and damage evolution in composite laminates, are glass fibers, carbon fibers, and aramid fibers. The latter are aromatic polyamides and are known in the industry by their commercial names, Kevlar and Twaron [1]. Glass fibers represent the reinforcing element of primary importance for polymer composites, a characteristic attributed to them due to their remarkable mechanical performance, advanced electrical insulation properties, and high resistance to humidity absorption [10]. There are multiple types of glass fibers, each designated by a different letter, including E-glass, AR-glass, C-glass, D-glass, E-CR-glass, and others [9]. Each of these glass fiber types is briefly described in a relevant section of this article. There are three parameters of interest regarding the influence of glass fibers on the behavior of composite laminates under various loading conditions, whose well-controlled variation allows for customization of their mechanical properties. Specifically, these parameters are the fiber content, fiber type, and orientation of fiber [10].

The effects of using woven fabrics instead of mats or unidirectional fiber cross-plies when investigating the behavior of composite laminates subjected to various mechanical loads, including low-velocity impact, were intensively studied [10,11]. The fundamental difference between a woven fabric and a mat resides in the production method. A mat is fabricated from either short or long fibers which are technologically bonded together in an irregular pattern. In contrast, when a woven fabric is manufactured, the fibers are interlaced to create different patterns, such as plain weaves, twill weaves, basket weaves or satin weaves. Woven fabric laminas aim to limit the propagation of the impact damage by reducing the failure caused by interply delamination [12]. Also, unlike composite materials containing layers reinforced with unidirectional fibers, woven fabrics with warp and weft yarns provide enhanced strength and stiffness in these two mutually perpendicular directions [13].

The necessity for developing high-strength materials, guided by the variety of loading conditions that an engineering structure must withstand during its service life, directed the researcher’s judgment towards creating hybrid composite materials. These laminated composites consist either of multiple types of woven fabrics made from synthetic fibers inserted into a polymeric matrix or of a single type of fiber representing the reinforcing element for resins incorporating nanofillers, such as graphene nanofibers, carbon nanofibers, or nanotubes. The mechanical behavior of hybrid composite materials is a result of the synergistic interaction of the properties of each distinct component. Thus, the customization of materials within the framework of manufacturing advanced composites adapted to the requirements of industrial applications is accomplished through the controlled variation in each constituent. Moreover, the regulation of the design parameters of hybrid materials, such as fiber types, the chemical characteristics of the matrix (which can be thermoplastic or thermoset), stacking sequence, and fabrication techniques, enables the manufacture of materials characterized by unique properties.

In the impact evaluation of the composite laminates, performed by analytical methods or experimental testing, aramid fibers have distinctive elastic and mechanical properties, which support their utility in engineering applications where the structure must withstand loads, sometimes under a dynamic loading regime. Aramid fibers have good specific properties, as a result of their low specific gravity. A remarkable characteristic of aramid fibers is their excellent tenacity, making these fibers serve as the primary reinforcing element in the manufacturing process of bulletproof vests [1]. Other properties that emphasize their widespread use are their high modulus, low elongation, reduced thermal conductivity, and corrosion resistance [14].

However, composite laminates reinforced with aramid fiber possess certain constraints generated by their interaction with the service environment. The critical limitation of this type of material is its difficult adaptability to environmental conditions with a degrading effect upon the constituents, i.e., the fibers and the matrix. Most polymer matrices exhibit lower compatibility and adhesion at the interfacial boundary with the Kevlar fibers. This result is reflected in reduced interlaminar shear strength and susceptibility to delamination initiation [15]. The effect generated by the long-term exposure of the material to UV radiation or extreme temperatures represents a limitation that affects the broadening of the application fields of Kevlar fiber-reinforced composite laminates. Compared to their tensile strength, the capability of aramid fiber-reinforced composites to withstand compressive loads is estimated to be 15–20% lower, which imposes restrictions on the load types that the material can sustain without damage [16].

This study is devoted to presenting the performance of composite materials reinforced with synthetic fibers, such as glass fibers, Kevlar fibers, and their hybrid configurations, when subjected to impact loading. Essentially, the article describes the effects of the complex correlations between impactor geometry, ambient conditions, and the design pattern of composite laminates on the materials’ response to withstanding the stresses generated under simple or multiple impact loading conditions. In Section 1, a framework is presented regarding the advantages of composite materials and the necessity for customized materials when dealing with impact-loaded structures. Section 2 outlines general theoretical aspects regarding impact loading. Section 3 and Section 4 detail the effects of the manufacturing parameters (stacking sequence of the layers, fiber type, laminate thickness), environmental factors (temperature or UV radiation), and testing parameters (such as projectile nose shape) on the mechanical behavior of Kevlar fiber-reinforced composites and glass fiber-reinforced composites when subjected to impact loading. Section 5 emphasizes the mechanical properties of the vegetable fibers (flax, hemp, and jute), the impact performance of the vegetable fibers-reinforced composites under hygrothermal aging, as well as the effects of manufacturing parameters on their impact behavior. In Section 6, the effects of the hybridization of Kevlar fibers or glass fibers with vegetable fibers on the impact performance are presented from a sustainable perspective. Finally, Section 7 outlines a few conclusions based on the presented study, together with some recommendations for further research directions.

## 2. General Aspects on Impact Loading

The theory invoked in the scientific literature, related to the field of system dynamics, defines the impact as a complex physical phenomenon encountered in many mechanical systems performing relative motion [17]. Fundamental aspects pertaining to the framework of impact between two or multiple bodies are related to the bodies’ surface geometry, their material properties, and the constitutive models which mathematically define the interaction between them [18].

**Figure 1:**
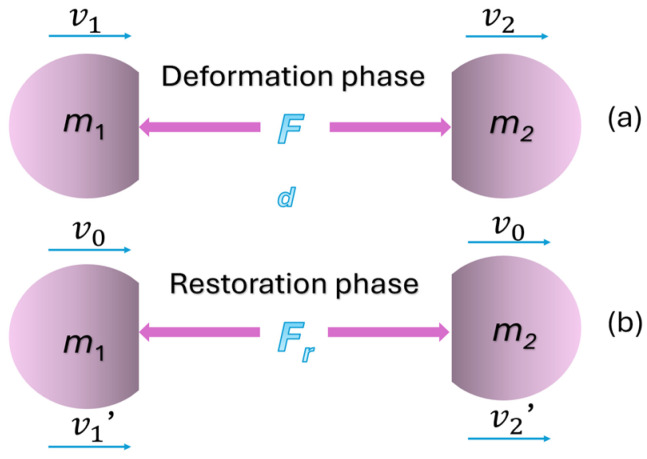
Direct impact between two spheres: (**a**) Deformation phase; (**b**) Restoration phase. (Adapted from [19]).

The simulation of the impact loading, from a theoretical point of view, implies modeling the contact between the impacted body and the impactor. Conceptually, contact can occur at a single point or at multiple locations, depending on the impactor surface geometry. A notion of paramount significance is the restitution coefficient, whose values lie in the interval [0, 1] and indicate either the elastic or plastic nature of the impact. Thus, if the impact is completely elastic, which means that the entire impact energy is dissipated into the system, the coefficient of restitution (COR) takes the unit value [19]. The normal restitution coefficient is dependent on the geometry of the impacted bodies, their materials, and the collision velocity. The basic impact between two spherical bodies is illustrated in Figure 1. Two stages, namely deformation and restoration, can be observed during the impact phenomenon. In the deformation period, spheres having mass $m_{1}$ and mass $m_{2}$, respectively, have distinct velocities $v_{1}$ and $v_{2}$. When the deflection of the bodies caused by the deformation force $F_{d}$ reaches its maximum value, both spheres have the same velocity $v_{0}$. When the contact between the spheres ceases, in the restoration period, each sphere tends to regain its initial position under the force $F_{r}$, with the velocities ${v_{1}}^{\prime }$ and ${v_{2}}^{\prime }$.

Tuo Li et al. [20] performed an experimental study regarding the contact-impact problem between two spherical particles, exploring the influence of spheres’ material and diameter, as well as their collision velocity on the COR. The experimental data proved that the effect of increasing spheres’ diameter and their collision velocity is represented by the reduction in the COR because of the increased energy loss. The common definition of COR is expressed by Equation (1), meaning the ratio between the relative velocity of separation and the relative velocity of approach [19,20]. It must be noted that the notation of the COR varies from one scientific reference to another.

(1) $$COR=-\frac{{v_{1}}^{\prime }-{v_{2}}^{\prime }}{v_{2}-v_{1}}$$

**Figure 2:**
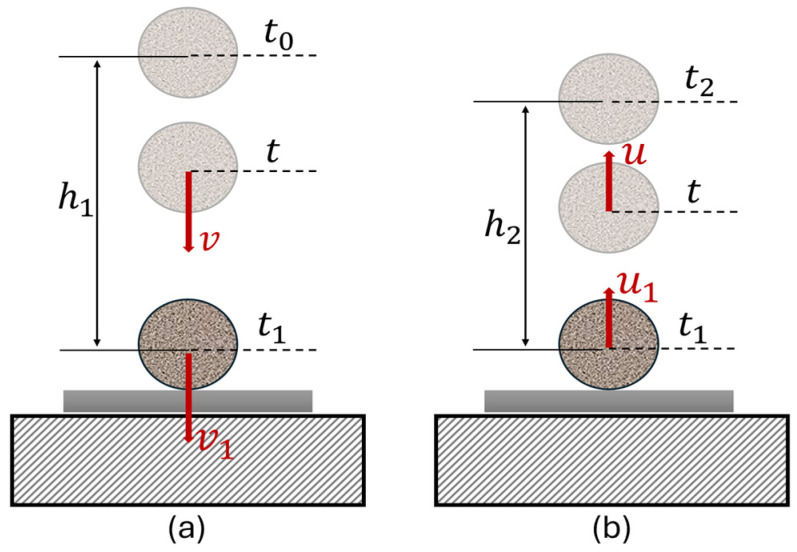
Impact between a sphere and a plate: (**a**) pre-collision; (**b**) post-collision. (Adapted from [21]).

Another case of a mechanical system which warrants consideration regarding the analysis of low-velocity impact (LVI) on composite laminates is an elastic ball freely dropped from an initial height. I. F. Moholea [21] analyzed the COR in this case, considering that the ball rebounds after impact. The formula used for determining the COR is expressed by Equation (2), where $u_{1}$ and $v_{1}$ are velocities and $h_{1}$ and $h_{2}$ represent heights at different time steps, as schematically shown in Figure 2.

(2) $$COR=\frac{u_{1}}{v_{1}}=\sqrt{\frac{h_{2}}{h_{1}}}$$

In the scientific literature, there are multiple theoretical models for expressing the contact between bodies with elementary geometry, such as two spheres, two cylinders, or a sphere and a plate [22]. The methods for finding solutions to applications that address the impact problem in the general case, excluding the facile examples previously listed, require the formulation of certain models to accurately describe the contact between the surfaces of the bodies. As a general classification, the two main approaches are penalty models and non-smooth models. The models in the first category are also known as contact-force, compliant, or regularized models. The approaches considered in the second category can be identified as methods based on geometrical constraints or discontinuous models [23]. A characteristic of penalty models is the dependence of the contact forces on the local properties of the contacting bodies [18]. Moreover, these forces are expressed as functions that have the penetration between bodies as the defining parameter, which provides computational efficiency [24].

The first constitutive law, considered the simplest model, was proposed by Hooke in 1661 [18]. A formulation that preceded a variety of subsequent solutions was defined by Heinrich Hertz, subsequently improved by Hunt and Crossley [24]. The Hertzian contact law is given by Equation (3):

(3) $$F_{N}=K\delta^{n},$$

where K is the contact stiffness parameter and n is the polynomial order.

Hunt and Crossley formulated Equation (4), which expresses the relationship between the force $F_{N}$ and the deformation δ along the normal direction, as a function of the hysteresis coefficient χ related to the damping effect. This equation served as a foundation for a variety of subsequent mathematical models used to describe the contact between surfaces.

(4) $$F_{N}=K\delta^{n}+\chi \delta^{n}\dot{\delta }$$

Over time, various formulations for the hysteresis coefficient χ were developed, leading to the modeling of particularized constitutive laws. E. Corral et al. [24] summarized in a comprehensive table published in their paper the mathematical formulations of different constitutive laws extracted from scientific literature and used to model the contact between bodies.

**Figure 3:**
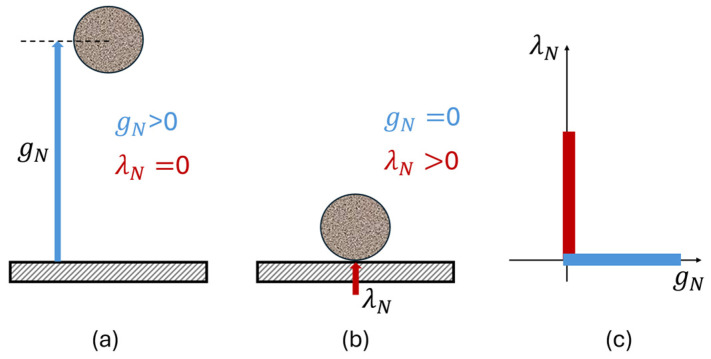
Impenetrability condition between two bodies in contact: (**a**) Open or inactive contact; (**b**) Closed or active contact; (**c**) Complementarity corner. (Adapted from [18]).

The alternative method for approaching contact-impact problems is the use of discontinuous models. The fundamental principle of these types of models implies the impenetrability condition of rigid bodies (Figure 3). This condition was formulated by Signorini-Fichera [18,25], and it is expressed by Equation (5):

(5) $$g_{N}\geq 0,\;\lambda_{N}\geq 0,\;g_{N}\lambda_{N}=0$$

where the product between the distance $g_{N}$ along the normal direction and the contact force $\lambda_{N}$ along the same direction must be null.

The causal relationship between impact loading and the propagation of stress waves, the initiation of deformation within the elastic or plastic regime, and the energy dissipation within the composite material system determined the experimental and theoretical studies of various composite laminates subjected to impact loads induced by impactors with specific properties.

## 3. Mechanical Behavior of Kevlar Fibers Reinforced Composites Subjected to Impact Loading

### 3.1. Mechanical Properties of Kevlar Fibers

Aramid fibers are known in the scientific literature for their remarkable strength and stiffness properties, which allow them to be used as reinforcing elements for polymeric matrices in the material design process intended for load-bearing applications.

The mechanical characteristics of Kevlar fibers are influenced by the fiber’s physicochemical state defined at the molecular level. There are two parameters that are of cardinal significance for describing their mechanical behavior, specifically the polymer chain rigidity and the crystallinity [26]. Some of the key mechanical properties extracted from research studies are presented in Table 1.

Moreover, Kevlar fibers exhibit great damping properties, allowing them to absorb vibration energy, which is a significant feature when assessing their dynamic loading behavior. Experimental evidence emphasizes that a higher damping ratio implies a reduced vibration amplitude and a higher energy dissipation [33].

### 3.2. Effects of the Parameters Related to Manufacturing Process on the Impact Behavior of Kevlar Reinforced Composites and Kevlar Hybridized Configurations

**Figure 4:**
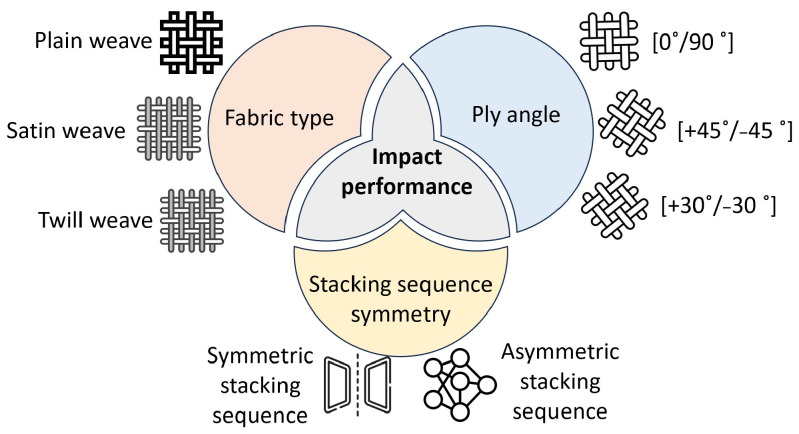
Laminate characteristics affecting the impact properties (Flat emoji icons from [36]).

The mechanical behavior of a composite material consisting of two different phases is partially dependent on the reinforcement properties and partially influenced by the physicochemical state of the matrix [34]. The impact behavior of composite laminates, and implicitly the performance of Kevlar-reinforced polymers, is strongly dependent on certain parameters related to the manufacturing process, including fabric structure, fiber thickness, number of laminas, orientation of the laminas, and the symmetry or asymmetry of the stacking sequence [35]. Figure 4 is suggestive regarding the composite laminate characteristics that affect the impact properties of the composite materials reinforced with continuous fibers.

Reinforcing the composite laminates, intended to withstand off-axis impact loads, with laminas whose warp and weft yarns are oriented at 0° and 90° with respect to the global coordinate system of the composite material, involves the transfer of impact energy only along the principal directions of each lamina. To overcome the constraint imposed by this fiber orientation and to enhance the capability of the laminate to absorb and dissipate impact energy, multidirectionally oriented fibers, such as [0°/22.5°/45°/67.5°], are preferred [37].

The study conducted by K. Czech and M. Oleksy [38] addressed the analysis of the effects of the number of laminas, ply orientation, and weave structure on the impact performance of Kevlar-reinforced epoxy polymers. The results, which are presented in Table 2, revealed that laminates reinforced with the highest number of twill fabric laminas (12) exhibited an energy absorption value 44.63% higher compared to the samples reinforced with the same number of laminas, but having plain weaves.

A comprehensive study regarding the morphology of core-spun compound (CSC) yarns and their ballistic impact behavior was conducted by D. Yang et al. [39]. The authors reported that the fabric woven from CSC yarns with Kevlar staple fiber demonstrated better impact performance than the compound yarn consisting of polyester staple fiber. Also, the experimental results proved that both fabrics could absorb a higher amount of impact energy than the pure Kevlar woven configuration. In the study conducted by Y. Liu et al. [40], which primarily addressed the effects of textile structure and impact energy on the capacity of thermoplastic composite laminates to withstand impact loads, the findings revealed that, among the three types of woven fabrics (three-dimensional orthogonal woven (3DOW), three-dimensional angle-interlock woven (3DAIW), and two-dimensional plain woven (2DPW) fabrics), the 3DOW structure demonstrated the highest strength under the single impact loading with energy levels of 10 J and 20 J. The three types of composites depicted specific failure modes when tested at high energy levels (10 J and 20 J). The 2DPW and 3DAIW composites failed by means of fiber breakage and delamination. Also, crack propagation was observed for the 3DAIW composite. However, the highest damage tolerance was observed in the material experiencing a tight interlocking effect, which is 3DOW. Regarding repeated impacts with an energy of 5 J, all types of fabric-reinforced composites exhibited similar behavior, while under an impact energy of 10 J, the 3DOW material demonstrated superior strength.

There are multiple impact regimes, depending on the impact velocity, namely low-velocity (0–50 m/s), sub-ballistic (50–500 m/s) and ballistic (500–1300 m/s) impacts [41]. Various studies were conducted on KFRPs regarding the response of the material to high-velocity impact loading.

Kevlar fabric is used to reinforce composite materials aimed at absorbing impact energy when subjected to ballistic tests, not only LVI. D. F. Leiva Palomera et al. [15] performed ballistic tests on neat Kevlar 29 fabric and on a polyurethane matrix reinforced with Kevlar 29 fabric having two different areal densities (400 g/m^2^ and 460 g/m^2^). They visually assessed the deformation of the impacted plates. Two types of ammunition were used, namely projectiles of 9 mm caliber and 5.5 mm caliber. The results showed that the 9 mm projectiles perforated all three tested plates, while the 5.5 mm projectiles were retained in each case. Their findings suggest that increasing the number of laminas with higher areal density leads to improved impact performance. This conclusion was also reported by K. Czech and M. Oleksy [38] in their study. A notable remark is that an increase in the surface density leads to a higher fragmentation level of the projectiles. This result can be attributed to the increased strength of the fabric offered by the higher thread count per unit area, preventing the projectile from penetrating the composite material.

C. Stephen et al. [42] performed high-velocity impact tests on neat Kevlar fabric reinforced epoxy matrix and on specimens reinforced with Kevlar, carbon fibers, and glass fibers in different stacking sequences. The results related to the neat Kevlar-reinforced specimen, consisting of 15 laminas, emphasized that the entire amount of impact energy was absorbed, with no penetration being visible. By doubling the thickness of the composite material and by increasing the impact velocity by 173.63%, the outcomes reported by Z. Li et al. [43] showed that the absorbed energy decreased by 136.18%, leading to shear and tensile failure of the Kevlar fibers.

The challenge of developing fiber-reinforced composite materials that demonstrate excellent impact behavior has been successively addressed. Hybridizing the filler phase of fiber-reinforced polymers can significantly enhance their mechanical characteristics and expand their application fields. Various studies addressing the effects of the stacking sequence, including the hybridization of composites reinforced with Kevlar fibers, were conducted in order to identify the optimal layer positioning to increase the impact performance at different velocities.

An interesting research study in which authors analyzed the effects of the reinforcement structure was performed by K. Czech et al. [44]. Their experimental work included an impact assessment section of an epoxy matrix reinforced with aramid, basalt, glass fibers, and the hybridized versions consisting of aramid-basalt and aramid-glass fibers. The best findings regarding the specimens, including aramid reinforcement, showed that the interleaving of aramid and basalt fibers achieved the highest impact strength of 115.9 kJ/m^2^ and an energy absorption level of 33.5%. Microscopic analysis revealed that the composite reinforced with Kevlar and basalt failed by inter-layer and intra-layer delamination of the basalt fabric.

The analysis performed by S. B. Loganathan et al. [45] addressed the effects of the asymmetric stacking of carbon and Kevlar-reinforced layers, and also of alternating laminas, on impact damage initiation and propagation. The results proved that composites having a Kevlar lamina on the top exhibited a 19% smaller damage area on the upper surface and a 28% smaller area on the lower surface for an impact energy of 8 J. When dealing with ballistic impacts, the placement of Kevlar fabric within composite laminates was also studied, especially when the thickness of the material becomes a parameter to be considered. S. Zhao et al. [46] stated in their study that thin composite laminates are more suitable for stacking Kevlar fibers at the bottom surface to fully benefit from high fracture elongation of the fibers. As the thickness of the composite laminate increases and the Kevlar lamina is placed on the bottom of the stack, the failure mechanism transitions from global bending of the composite to shear plugging, leading to a smaller damaged area.

Shifting to the sub-ballistic velocity category, V. Alagumalai et al. [47] assessed the impact response of Kevlar and E-Glass reinforced epoxy hybrid composites. During the fabrication process of the specimens, Kevlar plies were positioned only at the top and bottom of the composite laminate, with three different orientations (0°, 45°, and 60°), while the 16 glass fiber plies were stacked in the middle of the laminate. The experimental results demonstrated that composites with Kevlar oriented at 45° exhibited absorbed energy values 14% and 22% higher than the composites whose Kevlar plies were oriented at 0° and 60°, respectively. C. Stephen et al. [42] also analyzed the behavior of laminated composites reinforced with Kevlar and glass fibers subjected to high-velocity impact. The findings showed that, while keeping a similar material thickness of 3.2 mm, increasing the impact energy by almost 147% allowed the composite material to absorb the entire amount of impact energy, compared to the previous study. This behavior may be caused by the difference between the properties of the materials used.

Another experimental investigation regarding the effect of the stacking sequence in hybrid composite laminates reinforced with Kevlar, glass, and carbon fibers on the Izod impact performance was performed by S. Rout et al. The outcomes emphasized the positive effects on impact strength of combining two types of fibers, namely Kevlar and glass fibers, characterized by a high strain rate until failure, compared to the composite materials reinforced with all three types of fibers (Kevlar, glass, and carbon). Thus, the [G/G/K/K/K/G/G] stacking sequence proved the highest impact strength of 126 kJ/m^2^, compared to an impact strength of 80 kJ/m^2^ corresponding to the [C/G/K/C/K/G/C] stacking sequence [48]. Table 3 presents the results extracted from some research studies mentioned above, regarding the effects of manufacturing process parameters on the impact behavior of KFRPs.

In conclusion, manufacturing process parameters, such as the fabric weave type, number of laminas, thickness of the composite material, the location of the Kevlar ply in the stacking sequence, and its orientation, affect the impact behavior and damage mechanisms of the laminated composite, regardless of the impact velocity regime.

### 3.3. Effects of the Nanofillers on Impact Performance of Kevlar Reinforced Composites and Kevlar Hybridized Configurations

The impact requirements that composite materials must accomplish during their service life can be reached by integrating various types of nanofillers within polymeric matrices during the fabrication process. The addition of nanoparticles into a composite material system designed to withstand impact loads aims to enhance inter-yarn friction, energy absorption capability, and resistance to through-thickness penetration [50]. Several types of nanofillers are aluminum oxide (Al_2_O_3_), aluminum nitride or oxynitride (ALON), titanium dioxide (TiO_2_), silicon carbide (SiC), and zinc oxide (ZnO) [51,52,53]. The effect of the concentration of metallic nanoparticles inserted into the polymeric matrix is of paramount significance. After a threshold value, the positive contribution to impact strength is gradually reduced as the percentage of nanofillers increases. J. J. Joshua et al. [54] performed a study that quantified the influence of alumina powder on hybrid composites reinforced with Kevlar and basalt fibers. The analysis results indicated that the addition of a percentage of alumina powder higher than the threshold value led to the formation of agglomerates that determined the matrix breaking. Nanoparticles can be used not only by being mixed with a polymeric matrix, but also by being impregnated into Kevlar fibers, resulting in superior abrasion, wear, and tear strength [55].

Extensive research was performed on Kevlar-reinforced composite laminates regarding the effects of nanofiller addition on low-velocity or ballistic impact performance. Experimental test findings are presented in Table 4. The results demonstrate the positive effect of different types of nanofillers on the Kevlar-reinforced composite materials. Moreover, the impact performance and interlaminar shear strength of Kevlar-reinforced composite laminates depend on both curing temperature and curing time, whose optimal values were studied by B. Kumar and B. M. Rajaprakash, using the Taguchi L16 method [56].

Tailoring the friction between warp and weft yarns within the fabric through specific methods, such as the addition of nanofillers, can enhance the impact performance of the composite material reinforced with the aforementioned fabric. It was shown that mixing the polymeric matrix with SiO_2_ nanoparticles leads to improved adhesion of the matrix to the fiber surface. Also, coating Kevlar fabrics with silica particles leads to enhanced ballistic performance by increasing their stiffness and strength.

### 3.4. Effects of Temperature on Impact Performance of Kevlar Reinforced Composites and Kevlar Hybridized Configurations

Kevlar reinforced composite structures encounter temperature variations during their service life, depending on the field of application in which they are included. Also, there can be small temperature variations even when performing experimental tests on composite materials. The temperature gradient during testing of fiber-reinforced polymer composites affects the material toughness. Performing experimental testing under extremely low-temperature conditions leads to changes in the impact strength [57]. In contrast, A. Salehi-Khojin et al. [58] state that for a Kevlar-glass fiber hybrid laminate tested at 120 °C and a low impact energy level, the bottom surface cracking does not occur, but material bending and plastic deformation of the polymeric matrix are dominant. Table 5 summarizes the primary results and conclusions extracted from the scientific literature regarding the effects of temperature on the impact performance of Kevlar-reinforced laminated composites.

At negative temperatures, inter-yarn friction is higher when compared to high temperatures (above 50 °C). This characteristic limits the deformation of the yarns at low temperature, leading to a small, localized area where the fabric exhibited fiber breakage when the impactor hit the Kevlar fabric. In contrast, at high temperature, the friction between the fabric yarns is reduced; thus, the area where the fibers slip relative to each other is larger [59].

### 3.5. Effects of UV Radiation on Kevlar Fibers

The degradation of Kevlar fibers, as a result of the significant effects of the service environment characterized by extreme temperatures or rapid thermal fluctuations, together with the deteriorating effects caused by UV radiation, leads to a significant limitation regarding the stress level that the material can withstand, especially in the context of impact loading. Comprehension of the radiation shielding performance of specific composite materials allows the design of tailored products aimed at minimizing the radiation exposure of crew members who perform space missions. The amide-bond linkages of Kevlar fibers are sensitive to UV radiation, being easily cleaved under an irradiated environment [60].

Z. A. Oguz [61] demonstrated a decrease in the impact strength of the Kevlar Fiber Reinforced Polymers (KFRPs) and composite laminates hybridized with Kevlar fibers, after continuous exposure to UV radiation for 450 h. The author reports a partial improvement in the impact response of KFRPs after exposure to the UV spectrum for 900 h. This outcome is attributed to the reorganization of the macromolecular chains of the polymeric matrix. The same author described the findings regarding the effects of UV aging on the hardness properties of Kevlar fibers reinforced epoxy matrix materials. The analysis outcomes revealed that the hardness increased by 24.6% after exposure to UV radiation for 40 days, because of surface embrittlement and photochemical changes in the polymeric matrix [62].

Various methods aimed at enhancing the UV resistance of Kevlar fibers were explored, including sol–gel and hydrothermal treatments [63]. Moreover, in the context of impact energy absorption, shear thickening fluid (STF) and titanium dioxide (TiO_2_) fabric treatment proved to be remarkably beneficial for human protection equipment. The physical parameter of the fluid that affects the amount of energy absorption is viscosity, which fluctuates with the value of the applied external force. Also, TiO_2_ nanoparticles have remarkable UV absorption properties, thus increasing the radiation resistance of Kevlar fabric. Table 6 presents the variation in the absorbed energy as a function of UV irradiation time for specimens made of Kevlar fiber containing different weight percentages of SiO_2_ and TiO_2_. The results given in Table 6 are extracted from the study performed by D. Li et al. [60]. Analysis of the effects of UV radiation on the mechanical properties of Kevlar- reinforced composites used for firefighters’ protective clothing was addressed by S. Houshyar et al. The critical analysis results emphasized that after four days of UV exposure, the material was unable to withstand the mechanical loads to which it was subjected during the experimental assessment [64].

Expanding the application field, radiation shielding performance of Kevlar fabric was used to create an integrated solution for space missions performed by the International Space Station (ISS). In the study performed by L. Narici et al. [65], the behavior of three detectors was compared, one of them being unshielded and the others being shielded with two different areal densities, namely 5 g/cm^2^ and 10 g/cm^2^, of Kevlar and Polyethylene, under UV radiation conditions on the ISS. The analysis results demonstrated that dose rate ratios corresponding to the linear energy transfer (LET) ranges (30–50, 50–70, 100–150, 150–230 and 230–250 keV/μm) of the 10 g/cm^2^ Kevlar shielded detector are higher than those corresponding to the 10 g/cm^2^ Polyethylene-shielded detector with the following percentages: 20%, 10%, 5.55%, 4.87%, and 3.84%.

Concluding, UV radiation has a negative influence on the Kevlar fabric, resulting in undesirable mechanical performance after exposure beyond a threshold period. Fiber treatments are a current practice used for increasing the UV resistance of materials, especially in the context of the need for high impact strength. It was proved that the addition of TiO_2_ contributes significantly to the absorption of impact energy in tests performed with a knife impactor on the Kevlar plain weave fabric.

### 3.6. Degradation Mechanisms of Kevlar-Reinforced Composite Materials Under the Action of Environmental Factors

**Figure 5:**
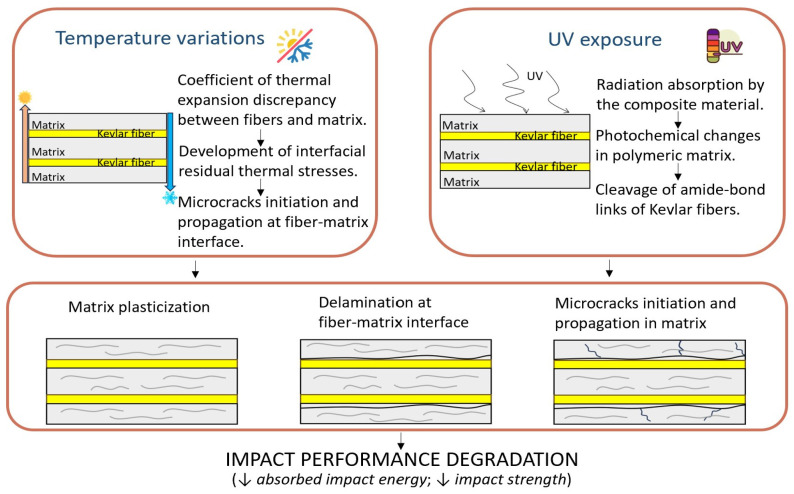
Degradation mechanisms of Kevlar/polymeric matrix composite materials caused by environmental factors and the effects on impact performance; the symbol ‘↓‘ indicates a decrease (Flat emoji icons from [36]).

Exposure of Kevlar-reinforced composite materials to destructive environmental factors determines coupled degradation mechanisms in the laminated composite structure. Summarizing what was presented in the previous Section 3.4 and Section 3.5, Figure 5 illustrates a schematic diagram of the damage mechanisms occurring within the Kevlar-reinforced polymeric composite materials subjected to either temperature variations or UV radiation. Matrix embrittlement, interfacial debonding or delamination, microcracks initiation and propagation affect the capacity of the composite material to absorb and dissipate impact energy. Kevlar fibers, like vegetable fibers, demonstrated an accelerated reduction in load-bearing capacity caused by moisture absorption, compared to other composites reinforced with synthetic fibers (glass and carbon fibers), under humid conditions.

### 3.7. Effects of the Projectile Nose Shape on Impact Performance of Kevlar Reinforced Composites

Projectile penetration through a material is a complex, transient phenomenon; thus, various studies have been conducted to assess the effects of the impactor nose shape on the material failure mechanisms [66]. The kinetic energy transferred by the impactor to the fabric-reinforced composite material target is absorbed by fiber yarns and the polymeric matrix.

Energy absorption mechanisms depend on the fabric type and the projectile nose shape. Scientific research studies addressed the investigation of deformation features of the Kevlar-reinforced polymers induced by different impactor geometries, such as conical, hemispherical or flat nose geometry, through experimental tests and numerical simulations. M. K. Dewangan and S. K. Panigrahi [67] evaluated the damage and penetration level of Kevlar plain fabric reinforced epoxy materials with variable thickness, subjected to impact with projectiles having conical and flat nose shapes. The main findings proved that sharp projectiles, with nose angle up to 60°, deformed rapidly and caused fiber wedging within the composite material. Sharp conical projectiles resulted in lower energy absorption by the target compared to impactors with higher nose angle values because only a few yarns were broken during the penetration of the composite plate. In contrast, for nose angles between 165° and 180°, including a flat nose projectile, the energy absorption increased due to the shear failure of the yarns.

Applying the same research methodology, J. Shi et al. [68] characterized the behavior of 3D angle-interlock Kevlar fabric reinforced epoxy matrix materials subjected to impact with a projectile having conical, hemispherical, or cylindrical nose geometry. The authors reported that the dominant failure mode induced by the conical impactor on the composite laminate was fiber fracture. Also, the hemispherical impactor determined debonding between the fibers and the matrix, while the cylindrical one caused minor resin deformation. A recent study performed on plain weave Kevlar-reinforced Methyl Methacrylate (MMA) thermoplastic matrix revealed that a composite material consisting of 16 layers of Kevlar fabric impacted with a 0.38 lead round nose (LRN) projectile at a velocity of 300 m/s absorbed the entire transferred energy from the impactor, without specimen perforation. In contrast, when the same type of composite material is impacted with a 0.357 Semi-Jacketed Soft Point (SJSP) projectile at a velocity of 550 m/s, the specimen was perforated, and the residual velocity was equal to 440 m/s [69].

Overall, different types of impactor nose shapes determine the formation of various damage areas and produce distinct damage mechanisms through the thickness of the Kevlar-reinforced composite laminates. In addition, the values of residual properties of both the impactor and the target laminate vary along with the impactor geometry.

## 4. Mechanical Behavior of Glass Fibers Reinforced Composites Subjected to Impact Loading

### 4.1. Mechanical Properties of Glass Fibers

One of the most common types of fiber used as a reinforcing element for polymeric matrix is glass fiber. Glass fibers were the invention of the French scientist René Ferchault de Réaumur [70]. Being more versatile than carbon fibers and less costly, glass fibers have remarkable properties, including high strength and stiffness, flexibility, and resistance to chemicals [71].

The particular properties of glass fibers in the composite materials industry vary depending on their chemical composition. The main elements contained in the fiber structure are silica, soda ash, and limestone. Silica is a constituent intended for the formation of glass, while the latter two constituents serve to reduce the temperature corresponding to the melting point [70,72].

**Figure 6:**
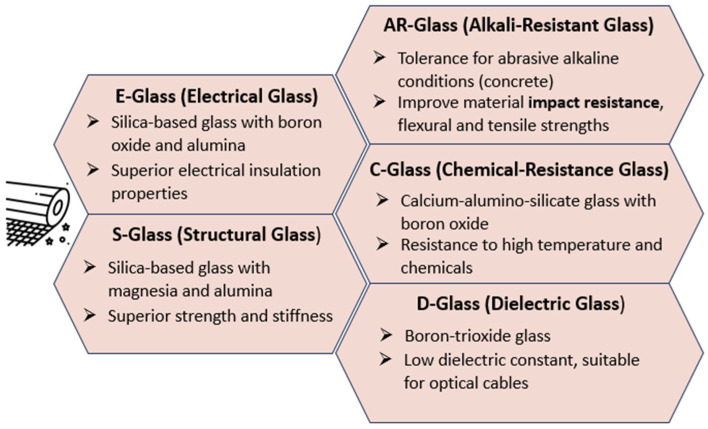
Glass fiber types and their characteristics.

In scientific literature, few significant categories of glass fibers can be distinguished, each of them having specific utilities. A concise classification of glass fiber types, together with their related characteristics, is illustrated in Figure 6. Among the glass fiber types, E-glass fibers are emphasized for their impressive electrical insulation properties, S-glass fibers are known for their remarkable tensile strength, while C-glass and D-glass give chemical resistance and dielectric performance. Regarding their behavior in withstanding dynamic loads, Alkali-Resistant (AR) glass fibers assign high impact strength and tolerance to an alkaline environment to the composite material they serve as reinforcements [73]. Table 7 presents the elastic and mechanical properties of the main types of glass fibers, reported in the research studies.

### 4.2. Effect of Parameters Related to Manufacturing Process and Glass Fiber Reinforced Configuration, on the Impact Behavior of Glass Fibers Reinforced Composites

Glass fiber reinforced polymers have superior impact strength compared to carbon fiber reinforced composite laminates, a fact which is attributed to the higher value of the strain-to-failure ratio of GRFP [79]. Aramid fibers possess impressive flexibility, having an elongation at break value within the range of 2.4–3.6% [80]. In Table 1 in Section 3.1, the maximum value of the elongation at break is 4%, reported for Kevlar fibers. Along with low density and high tensile strength, Kevlar fibers demonstrate the ability to form a tough, impact-resistant structure [81].

Mechanical properties of GFRP, implicitly its impact performance, depend on the parameters related to the manufacturing process, in the same manner as those of Kevlar fibers reinforced composite materials. The necessity to understand the effects of the variation in each parameter considered within the manufacturing process, especially the parameters which most significantly affect the impact strength of the glass fibers reinforced composite, has prompted the conduct of thorough experimental and numerical studies within the research community.

S. AlOmari et al. [82] performed an interesting study where the effects of stacking sequence and resin types (such as epoxy, phenolic, and polyester resins) on the low-velocity impact properties of glass fibers, carbon fibers, and their hybridized configurations were investigated. For this study, the performance of glass fiber reinforced polymers was of primary interest. Table 8 summarizes the resulting values of the absorbed energy of each sample containing only glass fibers as reinforcement. C. Kaboglu et al. [83] examined the effect of the curvature of composite materials manufactured with two different stacking sequences on their impact strength. The analysis outcomes revealed that the impact strength of the specimens decreased as the curvature increased. Considering the data shown in Table 8, it must be noted that for approximately the same thickness of 5 mm and the same impact energy of 150 J, both plain woven E-glass fabric reinforced epoxy composite and unidirectional glass fibers reinforced epoxy composite absorbed about 50% of the impact energy. Nevertheless, the study performed by J. W. Wong et al. [84], addressing the effects of both fiber structure and stacking sequence in glass fibers reinforced polymers on the impact behavior, concluded that bi-axial Glass Fibers Reinforced Polymer (GFRP) dissipated a higher amount of impact energy than unidirectional laminas reinforced composites. A. I. Ra’eis et al. [85] investigated the influence of five stacking configurations of pultruded glass fibers reinforced composites on the energy absorption capacity during impact loading. The key findings showed that inserting a larger number of laminas and alternating 0°, 45°, and −45° fiber orientations led to improved impact energy dissipation. Regarding hybridized configurations, the research performed by R. Maier et al. [86], which addressed the effects of the hybridization of glass and carbon fibers in composite materials on low-velocity impact performance, concluded that the absorbed energy values were higher for the specimens manufactured by alternating glass fiber laminas with carbon fiber laminas than those corresponding to the samples manufactured by alternating groups of glass fibers with groups of carbon fibers. The authors attributed this finding to the high stiffness of carbon fibers, which creates a mechanism for efficient impact energy absorption when the fibers are positioned in high-stress areas. It is concluded that cross-ply laminated composites are advantageous in terms of the perforation energy threshold.

### 4.3. Effect of Temperature on Impact Properties of Glass Fibers Reinforced Composites and Glass Fiber Hybridized Configurations

Exposure to a service environment with temperature fluctuations or extreme temperatures can cause morphological modifications, which influence the impact behavior of glass fiber reinforced composite materials. In addition to the temperature level, exposure time has a significant effect on the impact performance of the laminate [88]. Low temperature generates matrix embrittlement, while high temperature enhances the ductility of the continuous phase [8,89]. Impact loading at low ambient temperatures produces rapid crack initiation, which leads to large-area damage in the material [90]. A. M. Amaro et al. [91] state that the effects of decreasing temperature on the impact behavior of the composite materials regarding the damage propagation are comparable to the outcomes generated by increasing the impact energy value.

The authors B. Vieille and A. Coppalle [92] performed a study that assessed the thermal degradation induced by a kerosene flame on the impact behavior of the hybrid composite material based on Polyether Ether Ketone (PEEK) reinforced with both glass fibers and carbon fibers. The primary results of their study regarding the effects of testing temperatures, i.e., room temperature (RT) and 150 °C, on the impact performance of the earlier mentioned hybrid composites, are presented in Table 9. The authors concluded that the permanent indentation of the specimens subjected to fire exposure increased by approximately 40% for each of the tested impact energies, and the peak force decreased by 65% compared to the samples that were not subjected to thermal degradation.

### 4.4. Effect of Hygrothermal Aging on Impact Performance of Glass Fibers Reinforced Composite Materials

Hygrothermal aging is defined as the result of the concurrent action of temperature and moisture acting on the composite structure. Water molecules possess a strong affinity for hydrophilic components existing in various types of polymeric matrices [93]. Water diffuses through the macromolecular chains of the matrix or through microvoids caused by the manufacturing process, and determines the expansion of the polymer constituents, leading to weak intermolecular bonding forces. This phenomenon is referred to as the plasticization of the material [8]. Water absorption depends on the polymeric matrix type. A notable characteristic of epoxy resin is its high moisture diffusivity, which results in volumetric expansion [89].

D. Wang et al. [94] performed an analysis that addressed the investigation of the damage mechanisms of glass fiber-reinforced epoxy vinyl ester and unsaturated polyester composite materials subjected to a hygrothermal environment. The authors concluded that temperature and moisture have strong negative effects on composite laminates containing unsaturated polyester matrix, affecting both the appearance and the macrostructure of the material. An interesting experimental study regarding the impact behavior of T-joints after being subjected to hygrothermal aging was conducted by P. You et al. [95]. The primary results showed a significant reduction in both impact strength and impact force with increasing aging time and temperature values. Specific results extracted from their analysis are presented in Table 10. Degradation of the composite material reinforced with glass fibers resulting from prolonged exposure to high temperature is characterized by vertical microcrack initiation and propagation and a clearly visible dent after impact testing. As illustrated in Table 10, the displacement corresponding to the sample aged for six weeks and impacted with an energy of 17 J is 73.65% higher than that of the same material aged for the same period of time, but subjected to an impact energy of only 9 J.

M. Rabough et al. [96] performed a numerical analysis regarding the hygrothermal effects, i.e., 289 days at 50 °C with 95% relative humidity, on the impact performance of S-glass fibers reinforced polyester composite materials, considering three different impact energies (9 J, 13 J, and 18 J). The simulation results indicated that the maximum value of the contact force decreased as the moisture concentration increased. The authors concluded that water absorption has a beneficial effect on the impact force response until the saturation concentration, when the moisture causes material degradation. H. Li et al. [97] assessed the behavior of GFRP aged in two different environments, namely tap water and artificial seawater at 60 °C for 1200 h, and subjected them to multiple impacts. Analysis outcomes revealed that the total absorbed energy exhibited a decrease of 16% and 32% for the GFRP composites immersed in tap water and in seawater, respectively.

Compared to carbon fiber-reinforced composites, materials containing glass fibers exhibit greater changes in their elastic properties after hygrothermal aging. An experimental study performed by S. Yalçinkaya et al. [98] revealed that GFRP specimens immersed in seawater for 30 and 60 days exhibited a decrease in the value of the modulus of elasticity of 7.015% and 11.53%, respectively, compared to the specimens that were not subjected to aging. Also, the authors concluded that the visual changes corresponding to GFRP were more pronounced than the features determined through microscopic examination of CFRP.

### 4.5. Degradation Mechanisms of Glass Fibers Reinforced Composite Materials

**Figure 7:**
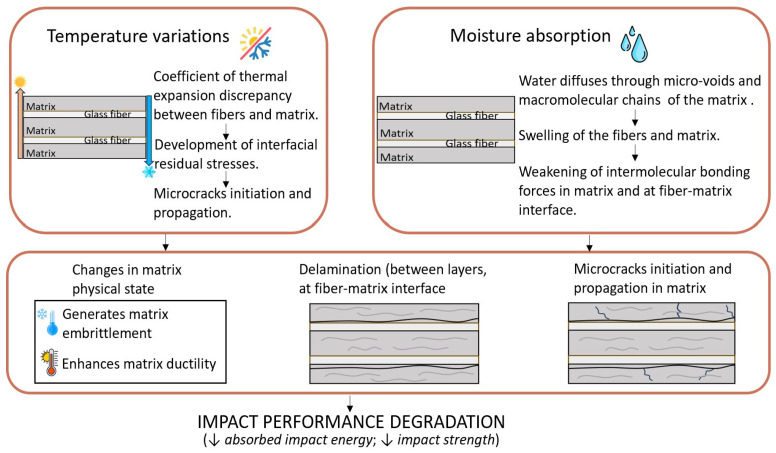
Degradation mechanisms in glass fibers/polymeric matrix composite materials caused by environmental factors and their effects on impact performance; the symbol ‘↓‘ indicates a decrease (Flat emoji icons from [36]).

Compared to Kevlar fibers, glass fibers possess higher resistance to thermal fluctuations and exhibit excellent behavior under humid environmental conditions. However, composite materials operating in a service environment with extreme temperatures or in an atmosphere conducive to water uptake develop residual stresses at the fiber-matrix interface. Considering what was presented in Section 4.3 and Section 4.4, Figure 7 highlights the damage mechanisms occurring in the composite material structure, which is based on polymeric resin reinforced with glass fibers. The development of thermal stresses at the fiber-matrix interface, resulting from temperature variations and moisture absorption, determines molecular-level modification of the resin. Extremely low temperature causes matrix embrittlement, while high temperature enhances matrix ductility.

### 4.6. Effect of Projectile Nose Shape on the Impact Performance of the Glass Fiber Reinforced Composites

Similar to Kevlar-reinforced composite materials, research on the effects of the projectile shape on glass fiber reinforced polymers represented the theme of numerous studies which aimed at assessing the damage mechanisms induced by the penetration or perforation of composite specimens.

Residual velocity and damage of the composite material elements vary depending on the projectile nose shape. Using the results obtained from numerical analysis, M. Kumar et al. [99] mentioned that a flat projectile impacting a composite laminate consisting of eight layers with a 0°/90° orientation had the minimum exit velocity compared to impactors having conical or ogival nose shapes.

The degradation of the laminated composite materials subjected to the impact loading is strongly dependent on the projectile nose shape. Degradation is quantifiable through the number of penetrated laminas, the layer delamination mode, and the deformation of the bottom layer [100]. An interesting study was performed by C. Arivoli and K. Ramajeyathilagam [101] regarding the effects of the spherical and conical shape projectiles on the impact behavior of the flat and curved specimens made of glass fiber reinforced polymers. Table 11 presents significant results extracted from their study, from FEM analysis, regarding the variation in the absorbed energy as a function of both the initial velocity of the impactor and the impactor nose shape. The analysis outcomes revealed that the energy absorption capability has a higher value for laminated panels impacted by a spherical-shape impactor, compared to those obtained with an impactor having a conical nose shape and manufactured with a large radius of curvature.

## 5. Mechanical Behavior of Vegetable Fibers Reinforced Composites Subjected to Impact Loading

### 5.1. Mechanical Properties of Vegetable Fibers: Flax, Hemp, and Jute

Vegetable fiber reinforced composite materials are used in numerous engineering applications, such as the automotive, aerospace, civil engineering, and sports industries, due to their acceptable mechanical properties together with low density, low cost, impressive energy absorption capacity, and biodegradability [102,103]. Flax, hemp, and jute fibers demonstrated specific strength and specific stiffness characteristics similar to those of glass fibers [104,105]. Mechanical behavior of bast fibers is strongly associated with the chemical composition of the natural fibers. The chemical constituents existing in the fiber structure that have mechanical relevance are polysaccharides, such as chitin and cellulose, both exhibiting higher stiffness in the axial direction than in the transverse direction [34]. Pectin represents a category of heteropolysaccharides aimed at increasing the structural flexibility of the fibers [105].

**Figure 8:**
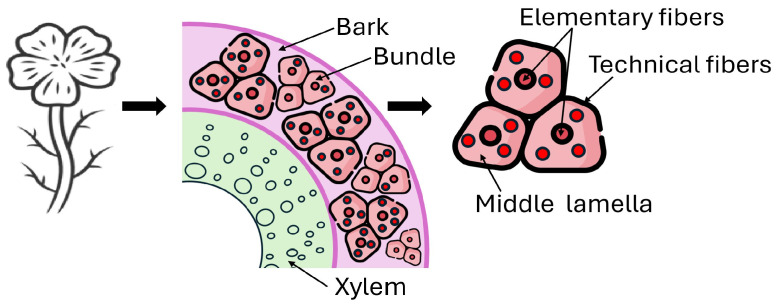
Morphology of transverse section of flax stem (adapted from [108]; flat emoji icons from [36]).

Flax is known in scientific literature as *Linum usitatissimum* [105]. Longitudinal fibers extracted from the stalk are among the oldest vegetable fibers used to manufacture woven fabrics, having higher strength, durability, and stability of properties under environmental fluctuations than jute fibers [106]. Flax, hemp, and jute, all of them being bast fibers, exhibit related internal structure. Figure 8 presents a general overview of the components of the flax fiber stem. Two concepts, frequently encountered in scientific articles related to the analysis of vegetable fillers, are elementary fibers and technical fibers. Technical fibers are grouped in bundles, and their characteristic dimension is their diameter, with values ranging from 30 μm to100 μm. Technical fibers contain multiple elementary fibers, having diameters between 10 μm and 20 μm [104]. Elementary flax fibers may naturally contain cell-wall defects which represent the tensile failure initiation points. These irregularities appear during the fiber processing procedure or are caused by the local misalignment of cellulose microfibrils [107].

Hemp is a plant belonging to the *Cannabaceae* family when referring to a botanical perspective. There are two cultivated species in Europe and Asia: *Cannabis sativa* L., used for fibers and seeds, and *Cannabis indica* Lam., cultivated for the production of narcotic substances [109]. Regarding the external morphological particularities, the root has multiple ramifications and can reach up to 1–2 m deep in the soil, the stem height can have values up to 5 m, and the stem diameter can range between 0.5 and 60 mm [109].

The hemp plant internal structure consists of two different types of fibers, i.e., bast fibers and the woody core parts, also called hurds or shives [110]. The woody part of the stem is categorized as agricultural waste, as it is removed during the processing of hemp fiber [111]. The percentage of long fibers, which can be used to create woven fabrics, contained within the hemp stalk, is on average 30%, the complementary percentage corresponding to woody shives [111]. Hemp fibers are predominantly used in the construction sector, encompassing multiple advantages, such as impressive thermal and acoustic properties, non-toxicity, energy efficiency, high tensile strength, and lightweight, when used as reinforcement for composite materials, representing an alternative solution to traditional building materials [112,113].

Jute is a vegetable fiber belonging to the *Corchorus* genus [114,115]. From a mechanical perspective, jute fibers possess impressive specific strength and stiffness, while having remarkable thermal and acoustic properties [114]. These characteristics are indirectly dependent on various natural factors, such as temperature, atmospheric humidity, soil fertility level, harvesting region, and harvesting time [116,117]. The particularities that directly affect the mechanical behavior of natural fibers, implicitly jute fibers, are their dimensions, i.e., length and diameter. There is a critical fiber length beyond which the mechanical properties decrease due to weak fiber-to-matrix adhesion and poor stress transfer, when the fibers are used as reinforcement in composite materials [118].

The mechanical behavior of a composite material containing two different components, namely the polymeric matrix and the filler, is proportionally dependent on the properties of each constituent. The geometry of the fibers, their dimensions, their humidity level, along with the physico-chemical state of the matrix, are factors that influence the composite material performance. The capability of the physical bonding between the fibers and the matrix to withstand the loads across the interface represents a factor of considerable importance when assessing the mechanical behavior of a composite material [119]. The difference in chemical composition between the lignocellulosic fibers and synthetic matrices leads to poor fiber-to-matrix adhesion. The hydrophilic character of cellulose, which exists in the structure of as-harvested vegetable fibers (such as flax, hemp, and jute), determines water absorption by the filler phase, contributing to the premature deterioration of the composite material [120]. Numerous treatments carried out on fibers for reducing the water uptake into the vegetable fiber structure were assessed. Optimizing the state of the intermediate phase of the composite material is fundamental for increasing its mechanical properties. Table 12 presents values extracted from the scientific literature for the mechanical properties of flax, hemp, and jute fibers, categorized based on values of fiber diameter. The diameter of the hemp stem is variable with the plant growth stage, having lower values within the flowering phase and increasing until the hemp reaches the maturity phase, which is appropriate for harvesting [121].

### 5.2. Effects of the Hygrothermal Aging on the Impact Performance of Vegetable Fibers (Flax, Hemp, and Jute) Reinforced Composite Materials

Natural fibers, regardless of their mineral, animal, or vegetable origin, are susceptible to deterioration caused by a humid environment due to their hydrophilic nature. Vegetable fibers analyzed in this review, i.e., flax, hemp, and jute, have chemical compositions containing cellulose, hemicellulose, lignin, and pectin. While lignin is the chemical component that allows for UV deterioration of vegetable fibers, hemicellulose contributes to water uptake within the fiber structure [126]. Fiber swelling, resulting from water absorption, can be reduced by increasing the fiber-to-matrix adhesion forces. In this regard, fiber surface chemical treatments are widely used to increase the hydrophobicity of the vegetable fillers.

Research studies regarding the influence of moisture absorption were conducted on flax fiber-reinforced composite materials. S.M. Vinu Kumar et al. [127] investigated the effect of salt water on the impact performance of the flax fabric-reinforced epoxy materials. The key findings demonstrated that for both dry and water-soaked samples, the impact strength increased with increasing fiber content. It must be noted that the flax fibers were pre-treated with alkali and trimethoxymethilsilane (ATS), and the specimens were immersed in water for 24 h.

Besides water immersion, the influence of humidity on the impact behavior of flax- reinforced composites can be assessed by applying a drying treatment to the fibers before proceeding to the manufacturing process. This interesting technique was used by R. Ciardello et al. [128] who dried the flax fabric in an oven at 60 °C for 8 h. They admitted that, after the heating cycle, the flax fabric laminas showed a weight loss of 6.1%. They used three different types of epoxy resin (Elium, IN2, and IB2) in the fabrication process, and each sample was subjected to a perforation test at an impact energy of 25 J. The key findings demonstrated that the maximum impact force was about 600 N for all resin types.

A comprehensive study was performed by Y. Shen et al. [129] regarding the temperature and moisture effects on the impact damage of flax-reinforced epoxy materials. Specimens consisting of 24 flax fabric laminas were immersed in deionized water for seven different time intervals (1, 2, 4, 6, 8, 12, and 16 weeks), then tested at impact energies between 1 J and 6 J (1 J, 3 J, 5 J, and 6 J). The key findings showed that before water immersion, the highest impact loads of 3450 N and 3140 N were recorded for samples tested at an impact energy of 6 J, before and after 16 weeks of immersion in water, respectively. Furthermore, morphological particularities emphasized that the larger displacement and damage area corresponded to the laminated composite material impacted at 6 J, while no delamination or fiber breakage occurred within the specimen tested at impact energies up to 5 J. After immersing the specimens in water for 16 weeks, the damage threshold limit decreased by 9%, and the micrograph of the damage initiation modes indicated that the amount of water absorbed by the samples led to flax fiber failure. It is remarked that the water absorption led to damage of the structure of the flax fiber, such as “the cell-wall peel off from the flax fiber”, in addition to the degradation of the fiber-matrix interface, according to the conclusions of the recent research [129].

Numerous experimental analyses were performed on hemp-reinforced composites to assess their impact performance after controlled exposure to aqueous environments. The study performed by Mieczyslaw S. et al. [130] addressed the effects of multiple water types, described by different salinity levels, on the impact performance of hemp fiber-reinforced polymers. The matrix used was a polyester resin based on DiCykloPentaDien (DCPD), particularly intended for use in yacht manufacturing. The primary results emphasized that the samples containing 11 chemically modified hemp fabric laminas demonstrated an impact strength 24.36% higher after immersion in fresh water than that corresponding to the glass fiber-reinforced polymer. The samples containing 11 unmodified hemp fabric laminas proved an impact strength only 7.05% higher than the value corresponding to GFRP. Also, the specimens consisting of 11 modified hemp fabric laminas soaked in water with a salinity level of 7.8‰ demonstrated an impact strength 68.98% higher than that of the GFRP samples tested under the same conditions.

Pursuing the same research path, some of the authors of the aforementioned article compared the properties of hemp fiber-reinforced composite materials with those corresponding to GFRP with the aim of determining a correlation coefficient between the two types of composite materials. Thus, Mieczyslaw S. et al. [131] concluded that it is appropriate to substitute conventional GFRP with environmentally friendly HFRP, based on the analyzed floating object.

P. D. R. Swain and S. Biswas [132] assessed the impact strength of jute fiber-reinforced epoxy matrix containing Al_2_O_3_ filler. The samples were immersed in distilled water for 10 days prior to the mechanical tests. Experimental evidence emphasized that increasing the fiber content results in a higher amount of absorbed water within the jute fiber structure. The authors attributed this behavior to the increase in the number of hydroxyl groups in the cellulose composition as the jute fiber content increases in the material. Also, the impact strength is unfavorably influenced by a humid environment. Thus, for the dry samples containing 30 wt.% jute fiber and 10 wt.% Al_2_O_3_, the absorbed energy was 1.9 J, while for the wet samples with the same percentages of reinforcing elements, the impact energy was about 1.75 J.

Experimental tests were performed by R. Gideon and D. Atalie [133] to explore the impact strength and the water absorption properties of jute and palm leaf reinforced hybrid composite material. Within the context of the Section 5.2, only the samples consisting of jute fibers are of interest, not the hybridized versions. The primary results emphasized that the composite material containing only jute fibers as the reinforcing element demonstrated the highest water absorption of 1.26% after immersion in water for 15 days at room temperature and the lowest impact strength of 19.02 kJ/m^2^. Table 13 presents the values extracted from this study, corresponding only to jute fiber-reinforced recycled polypropylene matrix. Also, Table 13 lists results regarding the effects of hygrothermal aging on the impact behavior of jute fiber-reinforced composite materials.

**Figure 9:**
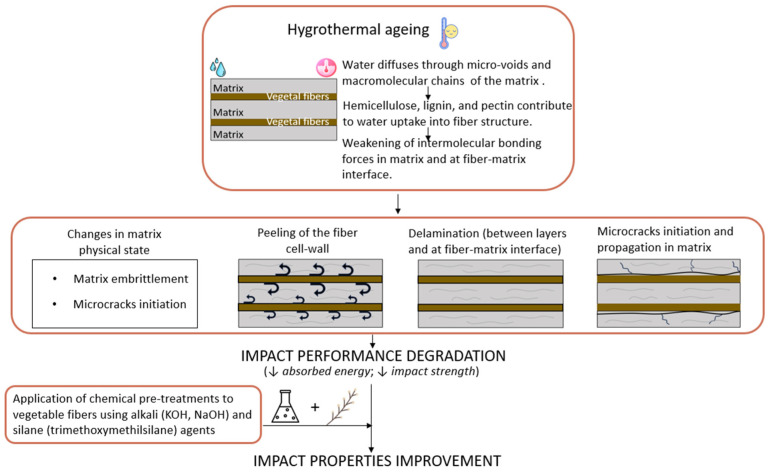
Degradation mechanisms in vegetable fibers/polymeric matrix composite materials caused by hygrothermal aging; the symbol ‘↓‘ indicates a decrease (Flat emoji icons from [36]).

The hydrophilic nature of vegetable fibers contributes significantly to moisture absorption within the fiber structure. To minimize the detrimental effects of fiber swelling, reflected in the impact strength of composite materials, the solution adopted by the scientific community was to increase the fiber-to-matrix adhesion by treating the reinforcing elements with various chemical substances, such as Al_2_O_3_, KOH, NaOH, or silane coupling agents. Figure 9 highlights the degradation mechanisms generated by hygrothermal aging within the composite material structure. Fiber swelling determines cell-wall peel-off, while matrix swelling causes interfacial debonding, microcracks propagation, delamination, and void formation induced by absorbed water evaporation.

### 5.3. Effects of the Parameters Related to Manufacturing Process and Vegetable Fiber (Flax, Hemp, and Jute) Reinforced Configuration, on the Impact Behavior of Vegetable Fibers Reinforced Composites

Parameters related to the manufacturing process, such as the stacking sequence or the reinforcing elements weight fraction, affect the impact behavior of vegetable fibers reinforced laminated composites. Either by hybridizing the vegetable fibers laminas with another type of natural reinforcements or with synthetic fibers, or by simply alternating the orientation angles of the lignocellulosic fibers laminas within the laminate stacking sequence, the impact strength of the material can be customized to meet the in-service requirements.

Numerous studies regarding the effects of alternating the stacking sequence of flax fabrics within the laminated composite materials were performed when subjected to impact loading. An interesting and extensive study was conducted by S. El Khoury Rouphael et al. [134] who tested unidirectional and cross-ply flax fibers reinforced composite materials under impact loading. The noteworthy section of their study implied the combination of unidirectional flax fiber-reinforced lamina with a mat binder consisting of two types of short flax fibers, conventional and fibrillated. All samples were tested at four different impact energies (3 J, 5 J, 8 J, and 11 J), and the key findings demonstrated that, for the unidirectional laminated composites, the absorbed energy has low values for the first two impact energies and an increasing trend for the last two energy values compared with the cross-ply composite materials. Another observation was related to the shape of the cracks formed within each of the materials analyzed. Thus, the unidirectional flax laminates exhibited longitudinal and transverse cracks, while for cross-ply flax laminates, the damage had a butterfly shape.

S. R. M. Rajendran et al. [135] studied the effects of hybridizing flax fibers with carbon fibers when creating laminated composites aimed to be tested under impact loading. From their study, only the average results corresponding to ±45° biaxially oriented woven flax-reinforced vinyl ester polymer material were extracted. Key findings showed that the absorbed energy of pure flax fiber-reinforced composite material had a value of 44.76 J, which is higher than the value of 33.52 J corresponding to the carbon hybridized configuration. Moreover, the rebound energy measured for the flax-reinforced polymer was only 0.91 J compared to the value corresponding to the carbon hybridized version of 12.67 J.

The textile structure influences the impact behavior of vegetable fiber woven fabrics. A. Alipour and K. Jayaraman [136] assessed the mechanisms of damage development in three different types of flax fabric (unidirectional, fine weave, and coarse weave) reinforced composites. The outcomes revealed that the fine twill weave has more unreinforced spots within the lamina; thus, the composite material subjected to impact loading dissipates a lower amount of energy compared to the coarse weave. The higher diameter fiber bundles, corresponding to the latter fabric, enable higher levels of energy absorption within the material.

R. S. Ramam and K. T. Padal [137] investigated the effects of both flax and hemp vegetable fiber lay-up sequences within the laminated composite materials subjected to impact loading. The authors studied the effects of lamina orientation angles (0°, 15°, 30°, and 45°) and their outcomes emphasized that the energy absorption trend was similar for both flax- and hemp-reinforced samples. However, the material containing hemp fibers showed slightly higher absorbed energy compared to the composites reinforced with flax fibers. Pursuing a similar research path, A. Vinod et al. [138] investigated the effects of the stacking sequence of the composite materials reinforced with jute and hemp fibers. The authors studied each type of pure fiber-reinforced polymer, as well as their hybridized categories. For Section 5.3, only the material containing pure hemp fiber or jute fiber reinforcement type presented interest. The key findings emphasized that the jute-reinforced composite material has the absorbed energy value of 6 J, which is higher than the value of 2.5 J corresponding to the hemp-reinforced epoxy material.

M. K. Singh and S. Zafar [139] performed a similar experimental analysis using a composite material containing three jute fiber laminas having the same orientation angle as those in the previously mentioned work [138], but the matrix was High-Density Polyethylene (HDPE). Their result regarding the impact behavior was emphasized by the impact strength value of 21.1 kJ/m^2^. Table 14 presents results extracted from the scientific literature regarding the effects of parameters related to the manufacturing process on the impact performance of vegetable fibers (flax, hemp, and jute) reinforced polymers.

It can be concluded that the stacking sequence, vegetable fiber texture in terms of weave fineness, and the hybridization of unidirectional fiber laminas with mat laminas affect the impact energy absorption capability of the vegetable fiber-reinforced composite materials. It was demonstrated that a composite material having laminas oriented at 0° absorbed more impact energy than the laminated composites with layers oriented at 15°, 30°, and 45°. This remark contradicts the conclusion extracted from the study performed by V. Alagumalai et al. [47], which highlights that composites reinforced with Kevlar fiber having the orientation 45° result in an energy absorption capacity higher than composites having laminas oriented at 0° and 60°. It can be noted that the impact behavior of natural fibers reinforced composites differs from that of the synthetic fibers reinforced polymers.

## 6. Impact Behavior of the Polymer Composites Reinforced with Vegetable Fibers Hybridized with Kevlar or Glass Fibers

### 6.1. Integration of the Vegetable Fibers into Hybrid Bio-Composite Materials: A Sustainable Perspective

To diminish the unfavorable effects generated by the manufacturing of composite materials reinforced with synthetic fibers, such as Kevlar and glass fibers, the scientific and technological communities adopted a new perspective on the fabrication process of the advanced materials. The novel strategy concerns the pollution reduction in the manufacturing process and the minimization of the resulting waste materials, emphasizing the sustainability of a more complex type of composite laminates, namely hybrid bio-composite materials.

The necessity of integrating sustainable materials into engineering structures represents the origin of the efforts conducted by composites designers and researchers, in accordance with the current trend toward the use of raw materials from renewable sources. The scientific community that operates within the field of civil engineering, especially in advanced materials science, is consistently and continuously exploring the alternatives that can demonstrate similar or improved performance compared to conventional materials, but with a low-carbon footprint. Composite materials incorporating vegetable fibers have become an attractive substitute solution not only for metallic materials, but also for concrete and steel structures [26]. Incorporating vegetable fibers into environmentally friendly materials promotes the reduction in the negative effects generated by the production and use of synthetic fibers. The use of panels made of conventional or hybrid configuration bio-composite laminates or in the form of a sandwich structure, whether they are flat or curved panels, allows for weight reduction while maintaining remarkable mechanical properties and their fail-safe nature, in this way avoiding oversizing the structure aimed to withstand dynamic loads [140]. Analysis addressing simple and multiple impact behavior of bio-composite panels must be performed in order to assess their performance under a set of established conditions, such as the environment and the loads imposed by their integration into helipad structures, helicopter structural parts or into civil applications, namely protective military shelters or roofing panels.

**Figure 10:**
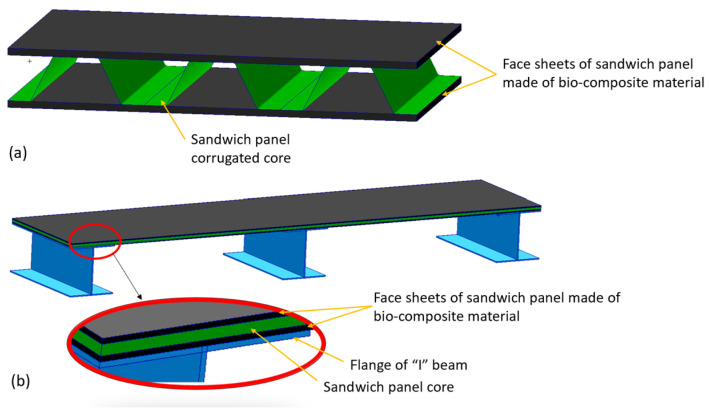
Suggestive applications of bio-composite laminates: (**a**) face sheets for sandwich panels with corrugated core; (**b**) face sheets for sandwich panels with full core.

Figure 10 presents two examples of possible applications of flat bio-composite panels, including face sheets for sandwich panels with a corrugated core or with a solid core, not only cellular ones, that can be used in the fabrication of civil construction structures. Ursache S. et al. [141] studied the effects of rubber core insertion on the low-velocity impact behavior of carbon-aramid reinforced composite materials. One of the main conclusions of their study emphasizes the delayed energy absorption recorded for the sandwich composite materials compared to the laminated composite specimens reinforced only with hybrid carbon-Kevlar fabric.

A possible application of curved bio-composite panels is the nose skin of a helicopter. The concept of embedding bio-based composite materials into the helicopter structure was embraced by the Airbus company [142]. Nevertheless, it must be noted that there exists the possibility of extrapolating the vegetable fiber types that can serve as reinforcement for bio-composite parts used in aeronautical structures. Inserting Kevlar laminates alongside vegetable reinforcement consistently increases the impact strength of the composite laminates.

### 6.2. Impact Performance of Kevlar Fibers Hybridized with Vegetable Fibers in Bio-Composites

Extensive research was performed on Kevlar hybridized with vegetable fibers reinforced polymers to assess their potential to meet the requirements imposed by applications subjected to out-of-plane impact loading. Hemp, flax, sisal, or jute fibers represent a few types of natural-origin reinforcement used as hybridization elements in the fabrication process of bio-composite materials. Experimental investigations of their impact performance are sometimes performed with the addition of various types of natural fillers, such as crab powder, palm shell, and coconut shell powder.

The study performed by H. Madhusudhana Reddy et al. [143] addressed the investigation of the hybridization effect of Kevlar with hemp and carbon fibers on the impact strength. The main results obtained for the two specimens analyzed showed that the absorbed energy for the sample with a 3 mm thickness was 12.5 J and for the one with a 5 mm thickness was 15.8 J. A. S. Ismail [144] approached the idea of evaluating the high-velocity impact strength of the interwoven carbon and Kevlar (CK) fibers hybridized with flax fibers reinforced epoxy containing bio-phenolic resin. The results emphasized that the composite material containing 25% flax fiber and 75% CK exhibited a reduction of 5.50% in the absorbed energy value compared to the neat CK-reinforced laminate. The same author conducted another study following a similar investigation direction, whose outcomes revealed a slightly different conclusion. For a specimen thickness of 3 mm, compared to the 6 mm value used in the previous study, the mixing ratio of 25:75 of flax to CK fibers demonstrated a 16.05% increase in the impact strength compared to the neat CK-reinforced composite materials [145].

B. A. Nagendram et al. [146] analyzed the impact performance of the composite laminates reinforced with Kevlar and hemp fibers. The addition of crab powder to the epoxy matrix is a distinctive element of their study. The analysis findings demonstrated that the sample with hemp laminas oriented at 60° absorbed the highest amount of energy under impact, namely 8.66 J, compared to the specimens including hemp laminas oriented at 45°, which showed the lowest value of the absorbed energy, 2.66 J. Pursuing the same research path, P. A. Thakare et al. [147] analyzed the impact behavior of Kevlar hybridized with both hemp and flax fibers to reinforce the epoxy matrix. The study outcomes revealed that for the four impact energies analyzed, namely 6 J, 12 J, 18 J, and 24 J, the corresponding residual energies were 1.42 J, 6.64 J, 8.80 J, and 15.40 J. Considering that the impact energy represents the summation of the absorbed energy and the residual energy, then the computed absorbed energy values for all impact energy levels were 4.58 J, 5.36 J, 9.20 J, and 8.60 J.

An interesting study was performed by S. P. Jani et al. The authors evaluated the influence of coconut and palm powders on the impact behavior of the Kevlar- and hemp-reinforced epoxy matrix. The primary conclusion emphasizes that adding natural powder to the polymeric matrix constitutes a benefit until a threshold content. In their study, when the filler content exceeded 5 wt.%, the impact strength of the samples exhibited a sharp decrease because of the insufficient resin quantity needed to impregnate the laminas [148].

Apart from hemp fibers, studies including jute fibers together with Kevlar as the reinforcing phase were performed. Gurusamy et al. [149] assessed the impact performance of Kevlar hybridized with jute fibers to reinforce the epoxy resin containing four different proportions of silica nanoparticles, which are intended to improve the mechanical and thermal properties. Two stacking sequences were studied, each of them including seven layers of alternating Kevlar and jute fibers. The outcomes revealed that the specimens consisting of Kevlar laminas oriented at 0° and jute layers oriented at 90° demonstrated better performance than the samples with the jute fibers oriented at 45°. Table 15 presents a few summarized results extracted from the studies presented above.

The effects of the incorporation of vegetable fibers into Kevlar-reinforced polymers, regarding the impact performance, are reflected in the energy absorption capacity of the material. Increasing the content of vegetable fibers until a threshold value leads to an improvement in the absorbed energy value. However, in most studied cases, a decrease in the impact strength is observed. This behavior is caused by the lower values of the mechanical properties of vegetable fibers compared to those corresponding to Kevlar fibers.

### 6.3. Impact Performance of the Glass Fibers Hybridized with Vegetable Fibers in Bio-Composites

Consistent research was performed on composite materials reinforced with a hybrid reinforcement configuration consisting of glass fibers and various types of vegetable fibers. Besides water absorption, thermal behavior, and mechanical properties, the impact performance of bio-composite laminates reinforced with glass and vegetable fibers generates increased interest within the research community.

T. Ozier et al. [150] highlighted in their study that adding partial bio-based content, represented by jute fibers, to glass fiber reinforced polypropylene leads to impact strength values which are close to those of Acrylonitrile-Butadiene-Styrene (ABS) and Acrylonitrile-Styrene-Acrylate (ASA). Their findings encourage the utilization of environmentally friendly materials as a substitute for the conventional composite materials used for manufacturing non-structural components in automotive applications. Various studies aimed at investigating the effect of the jute lamina position within the stacking sequence, the effect of wrapping jute fiber around a glass fiber core, or the influence of various chemical treatments, such as potassium hydroxide (KOH) and graphene oxide (GO), on the impact performance of the hybrid composite materials reinforced with glass and jute fibers were performed. Experimental results demonstrated that positioning the glass fiber laminas on the top and bottom surfaces of the laminate ensures better energy absorption than the specimens having jute laminas as the extreme layers [151,152,153]. V. S. Chinta et al. [154] investigated the effect of inserting one single layer of jute fabric at five different positions into the 14-laminas stacking sequence of a fan blade manufactured from glass fibers reinforced epoxy matrix. Compared to the original layup sequence, the experimental results showed that inserting one jute lamina in the eighth position led to a small reduction of 3.1% in the absorbed energy of the composite material.

An interesting study was performed by M. Islam et al. [155] regarding the effects of wrapping graphene oxide-modified jute fiber around a core of glass fiber on the impact performance of the composite materials obtained by inserting the novel hybrid fibers into an epoxy resin. The outcomes emphasized the significant contribution of the graphene coating to the enhancement of the adhesion between fibers and the matrix, leading to a specimen that demonstrated the highest impact strength of 85.56 kJ/m^2^. Following a similar research path, S. Ullah et al. [156] used potassium hydroxide to increase the bonding between the hybrid jute and glass fiber reinforcement and the epoxy matrix. The authors reported that KOH treatment removes non-cellulosic components, such as lignin and pectin, leading to improved adhesion between fibers and the matrix.

Flax fibers are also used as a hybridization element in recent studies, where energy absorption during low-velocity impact of bio-composites is analyzed. Waste glass fibers represent a performant tool to improve the impact performance of the flax composite laminates. Therefore, placing them within the structure of the material leads to a composite with higher impact strength, while maintaining its status as an environmentally friendly material [157].

T. Akshat et al. [158] performed both experimental and numerical investigations regarding the effects of combining flax fibers with glass fibers in four different stacking sequences. The main results revealed that increasing the number of glass fabric laminas enhances the mechanical performance, including energy absorption, of the composite laminates. Table 16 summarizes the main results discussed above regarding the effects of hybridization with glass fibers on the impact performance of vegetable fiber-reinforced composite materials.

Temperature also influences the impact performance of the glass fibers and flax fibers reinforced composite materials. M. A. M. Ahamed et al. [159] proved that at a temperature value of −10 °C, the composite with layers reinforced with flax fibers hybridized with glass fibers, positioned on the external surfaces, achieved the highest impact energy absorption level.

The solution adopted by incorporating lignocellulosic fibers into glass fiber reinforced polymer materials endorses the exploitation of the benefits conferred by natural origin reinforcing elements. Renewable resources represent a key component in defining a development path for engineering structures capable of withstanding mechanical loads and sustaining a sustainable future initiative.

## 7. Conclusions and Future Perspective

The primary purpose of this paper was to emphasize and centralize the results extracted from recent research studies regarding the performance of the composite materials reinforced with two categories of synthetic fibers, namely Kevlar and glass fibers, subjected to impact loading. It is noted that there were numerous studies that were oriented toward the analysis of the composite materials’ response under multiple mechanical loading conditions, such as axial loading, flexural loading, or vibrations, not only under impact loading. Thus, it was considered useful to provide a review dedicated exclusively to assessing the impact performance of the composite laminates reinforced with Kevlar and glass fibers in order to create a framework that is easy to visualize and interpret, summarizing the corresponding results. Before evaluating the influence of the various parameters (such as stacking sequence, fabric type, temperature, UV radiation, hygrothermal aging, and nose shape of the impactor) on the impact behavior of the composite materials reinforced either with Kevlar or glass fibers, a mathematical perspective describing the theoretical basis of the impact between bodies was approached. The impact performance of three types of vegetable fibers (i.e., flax, hemp, and jute) was also synthesized to highlight their behavior under humid environment conditions and the influence of both stacking sequence and fabric type on the impact energy absorption capacity.

Finally, the impact performance of the composite materials reinforced with vegetable fibers hybridized with Kevlar or glass fibers was presented to emphasize the impressive capability of renewable resources to be integrated into engineering applications. Based on the scientific literature analyzed, the following conclusions are highlighted:

- Considering the results shown in the references mentioned, it is noted that for the composite materials reinforced with Kevlar fabrics, subjected to low-velocity impact, the absorbed energy was in the range of 4.26–5.64 J, while this quantity was about 9.0–13.2 J for the composites reinforced with glass fabric, depending on the internal parameters of the impacted plates (matrix type, thickness, density of fabrics, number of layers). It was also remarked that the absorbed energy was lower, about 1.0–1.9 J, for composites reinforced with fabrics made of vegetable fibers (flax, jute, and hemp), leading to the hybridization of these kinds of fibers with Kevlar or glass fibers for the improvement of the energy absorption.
- For composite laminates reinforced with both categories of fibers, Kevlar and glass fibers, it was concluded that the energy absorption capacity is improved in the following ways: utilization of woven fabrics rather than unidirectional laminas; stacking sequence containing layers oriented at +45° or −45°, not just at 0° and 90° orientations.
- For composite laminates reinforced with vegetable fibers, the studies concluded that composite materials having laminas oriented at 0° absorb a higher amount of impact energy than the laminated composites with layers oriented at 15°, 30°, or 45°.
- Temperature variations, as well as the exposure time at a temperature of 60 °C (limited by the service temperature of the resin), affect the impact behavior of the Kevlar and glass fiber-reinforced polymers. It was observed that the impact energy generally decreased, while the maximum displacement increased during the impact test after aging of those materials at 60 °C. A low-temperature environment causes matrix embrittlement, while high temperature generates an increase in matrix ductility.
- Laminated composites reinforced with vegetable fibers are prone to water absorption due to the hydrophilic nature of the chemical components contained in their structure. This behavior affects the impact strength of the materials reinforced with lignocellulosic fiber. To overcome this issue, various chemical substances (SiO_2_, Al_2_O_3_, TiO_2_, and ZnO) are used to increase the adhesion between fibers and the matrix. After immersion for 3 months in water with a salinity of 7.8‰, the impact strength of the hemp/polyester composite increased by about 22–68%, depending on the number of hemp layers.
- UV radiation has a negative influence on the impact behavior of the Kevlar-reinforced composite laminates. After 168 h of exposure to UV radiation, the absorbed energy of the Kevlar/epoxy composite decreased by 60%. Performing fiber treatments represents a common practice for increasing the UV resistance of such composite materials. After the addition of shear thickening fluid (STF), SiO_2_ and TiO_2_, the absorbed energy of the Kevlar/epoxy composite, aged under UV radiation for 168 h, increased from 5 J to 14–16 J (depending on the quantity of each component), compared to the neat Kevlar-reinforced material.
- Hygrothermal aging of the glass fibers-reinforced composite laminates affects both appearance and internal structure of the material. After immersion for 1200 h at 60 °C in seawater, the absorbed energy of the glass fiber-reinforced composite decreased by 32% with respect to the value corresponding to the dry specimens.
- From a sustainable perspective, hybridizing Kevlar and glass fibers with vegetable reinforcements and applying fiber surface treatments to improve adhesion with the polymeric matrix offer the opportunity to partially substitute layers reinforced with conventional fibers with those reinforced with environmentally friendly fibers, maintaining a similar energy absorption capacity during the impact loading.

Most of the data reviewed in this paper are extracted from analyses performed on composite materials subjected to single impact loading. Moreover, considering the increasing interest at the global level for novel materials containing reinforcement fibers, originating from renewable sources, such as vegetable fibers, upcoming analysis, either experimental or numerical, should focus on the analysis of bio-composite materials subjected to multiple impacts. Two categories of multiple impacts can be distinguished, namely repeated impacts occurring in the same location or impacts with a larger number of impactors striking simultaneously at distinct, close locations. Within the second category, the effect of the nose shape geometry of each impactor on the energy absorption capability could represent an interesting research direction for future research.

## Figures and Tables

**Table 1:** Densities and tensile properties of different types of Kevlar fibers.

Fiber Type	Young’s Modulus E [GPa]	Tensile Strength σt [GPa]	Poisson’s Coefficient ν [-]	Elongation at Break ε [%]	Density ρ [g/cm^3^]	Ref.
Kevlar 29	70.00	2.90	0.37	4.00	1.44	[26,27]
	83.00	3.60	-	3.00	1.44	[28]
	68.90	3.20	-	3.90	1.44	[29]
	70.50	2.92	-	3.60	1.44	[30]
Kevlar 49	135.00	2.90	0.36	2.80	1.45	[26,27]
	131.00	3.60–4.10	-	2.80	1.45	[28]
	124.10	3.00	-	2.30	1.44	[29]
	112.40	3.00	-	2.40	1.44	[30]
	151.70	2.75	0.35	-	1.46	[31]
Kevlar 100	60.00	2.90	-	3.90	1.44	[26,27]
	60.00	3.00	-	2.90	1.44	[28]
Kevlar 119	55.00	3.10	-	4.40	1.44	[26,27]
	55.00	3.10	-	3.10	1.44	[28]
Kevlar 129	99.00	3.40	-	3.30	1.45	[26,27]
	97.00	3.45	-	3.40	1.45	[28]
Kevlar 149	143.00	2.30	0.30	1.50	1.47	[26,27]
	143.00	3.40	-	2.30	1.47	[28]
Kevlar 363 2S	59.60	1.02	0.30	-	1.40	[32]
Kevlar 802F	110.00	3.40	0.30	-	1.40	[32]

**Table 2:** Influence of the fiber type, number of laminas, and fiber orientation on the impact performance of the Kevlar-reinforced composite materials [38].

Property	P4	P8	P12	T4	T8	T12	P04	P08	P012	T04	T08	T012
No. of laminas	4	8	12	4	8	12	4	8	12	4	8	12
Weave type	Plain	Plain	Plain	Twill	Twill	Twill	Plain	Plain	Plain	Twill	Twill	Twill
Fabric stacking sequence	-	-	-	-	-	-	[A] *	[B] *	[C] *	[A] *	[B] *	[C] *
Impact parameters	Impactor diameter: 20 [mm]; impactor weight: 16 [kg]; impact velocity: 4.40 [m/s]; drop height: 1 [m]											
Absorbed energy [J]	5.10	18.20	36.60	11.80	34.00	74.00	6.10	20.50	52.20	10.30	41.30	75.50

* Abbreviation for the fabric stacking sequence used: [A] = [0°/22.5°/45°/67.5°]; [B] = [0°/22.5°/45°/67.5°/0°/22.5°/45°/67.5°]; [C] = [0°/22.5°/45°/67.5°/0°/22.5°/45°/67.5°/0°/22.5°/45°/67.5°].

**Table 3:** Influence of manufacturing process parameters on the impact performance of Kevlar-reinforced composite laminates.

Fabric Type	No. of Plies	Specimen Thickness [mm]	No. of Impacts	Impact Parameters		Abs. Energy [J]	IRI *	Remarks	Ref.
				E * [J]	v [m/s]				
(i) Nonhybridized Kevlar composites									
Low-velocity impact									
Plain weave	14	5.00	1	9.89	3.20	5.05	-	After the 3rd impact, the bottom surface presents a major matrix crack and severe delamination.	[49]
			3	9.89	3.20	5.16	-		
			5	9.86	3.20	4.26	-		
Stacking sequence: [0°]_14_; Matrix type: Epoxy; Impact method: Drop weight test system									
3D Orthogonal weave	4	3.30	1	10.00	1.68	5.64	0.77	For all three materials impacted at 10 J, fiber-breakage and delamination are visible.	[40]
3D angle-interlock weave	4	3.54	1	10.00	1.68	6.17	0.62		
Plain weave	4	3.31	1	10.00	1.68	6.49	0.54		
Stacking sequence: [0°]_4_; Matrix type: Epoxy; Impact method: Drop weight test system									
Sub-ballistic impact									
Plain weave Kevlar 129	15	3.20	1	226.24	201.00	226.24	-	No penetration or damage on the bottom face of the impacted plate.	[42]
Stacking sequence: [0°]_15_; Matrix type: Epoxy; Impact method: gas-gun apparatus									
Ballistic impact									
Plain weave Kevlar 49	27	6.40	1	-	550	95.96	-	Shear and tensile failure of Kevlar fibers were observed.	[43]
Stacking sequence: [K(0°)]_27_; Matrix type: Epoxy; Impact method: automatic projectile system									
(ii) Kevlar hybridized with carbon, basalt or with glass fibers composites									
Low-velocity impact									
Kevlar & carbon	6	2.15	1	8.00	1.97	7.84	-	Kevlar is the topmost layer. **	[45]
Kevlar & carbon	6	2.15	1	16.00	2.79	13.19	-	Kevlar is the topmost layer. **	
Stacking sequence: [K(0°)/C(0°)]_3_; Matrix type: epoxy; Impact method: Drop weight test system									
Kevlar & basalt	4	-	1	96.80	4.4	45.16	-	One Kevlar layer is at the bottom of the stack.	[44]
Kevlar & glass	4	-	1	96.80	4.4	8.24	-		
Stacking sequence: [B(0°)/K(0°)/B(0°)/K(0°)/]; Matrix type: epoxy; Impact method: Izod test									
Stacking sequence: [G(0°)/K(0°)/G(0°)/K(0°)/]; Matrix type: epoxy; Impact method: Izod test									
Sub-ballistic impact									
Kevlar & E-glass	16	3.00	1	115.60	170.00	44.80	-	Kevlar ply angle is 0°.	[47]
Stacking sequence: [K(0°)_1_/G(0°)_14_/K(0°)_1_];									
Kevlar & E-glass	16	3.00	1	115.60	170.00	58.70	-	Kevlar ply angle is 45°.	
Stacking sequence: [K(45°)_1_/G(0°)_14_/K(45°)_1_];									
Kevlar & E-glass	16	3.00	1	115.60	170.00	37.10	-	Kevlar ply angle is 60°.	
Stacking sequence: [K(60°)_1_/G(0°)_14_/K(60°)_1_]; Matrix type: epoxy; Impact method: gas-gun setup									
Plain weave Kevlar & EC9-glass	15	3.20	1	286.02	226.00	280.29	-	Pull-out of Kevlar fibers can be seen on the top and bottom faces of the impacted plate.	[42]
Stacking sequence: [K(0°)_5_/G(0°)_5_/K(0°)_5_]; Matrix type: Epoxy; Impact method: gas-gun apparatus									
Plain weave Kevlar & carbon	15	3.20	1	226.24	201.00	208.68	-	Damage to the bottom layer is localized at the impacted zone.	[42]
Stacking sequence: [K(0°)_5_/C(0°)_5_/K(0°)_5_]; Matrix type: Epoxy; Impact method: gas-gun apparatus									
Plain weave Kevlar & carbon	27	6.50	1	-	550.50	103.91	-	Circular perforation, Kevlar fiber breakage, and matrix failure are observed.	[43]
Stacking sequence: [K(0°)_20_/C(0°)_7_]; Matrix type: Epoxy; Impact method: automatic projectile system									

* IRI = Impact Resistance Index (IRI is defined as the ratio between rebound energy of the impactor and absorbed energy); E = impact energy. ** The topmost layer is towards the impactor.

**Table 4:** Effects of the nanoparticle addition on energy absorption capacity of the Kevlar reinforced laminated composites, and Kevlar hybridized configurations.

Fabric Type	Nanofiller Type	Nanofiller Content [wt. %]	Impact Parameters		Absorbed Energy [J]	Conclusion	Ref.
			Energy [J]	Velocity [m/s]			
Sub-ballistic impact							
Plain weave Kevlar 129	SiO_2_ (12 nm)	-	-	400.00	~24.00	Absorbed energy is 25% higher than the corresponding neat plain weave.	[55]
Stacking sequence: [0°/0°/0°/0°/0°/0°/0°/0°]; Matrix type: Epoxy; Impact method: FEM analysis							
Kevlar (warp) and basalt (weft)	Al_2_O_3_	5.00	184.68	224.33	39.34	Absorbed energy is 59.46% higher than that corresponding to the sample consisting of only 1% nanofiller content.	[54]
Laminate thickness: 3 mm; Matrix type: Epoxy; Impact method: high-pressure pneumatic accelerator							
Low velocity impact							
Kevlar—carbon hybrid	TiO_2_	5.00	-	3.50	10.10	Absorbed energy is 31.2% higher than the K-C hybrid composite without any orientation or nanofiller added.	[57]
Stacking sequence: [C(+45°)/K(−45°)/C(90°)/K(0°)/C(90°)/K(−45°)/C(+45°)]; Matrix type: Epoxy; Impact method: Izod							
Kevlar fabric mat (8 plies)	SiC & ZnO	0.75 & 1.75	-	-	60.00	Absorbed energy depends on the nanofiller weight percentage, curing temperature and curing time.	[56]
Stacking sequence: [0°/0°/0°/0°/0°/0°/0°/0°]; Matrix type: Epoxy; Impact method: drop weight test							

**Table 5:** Temperature effects on the impact behavior of Kevlar fabrics and Kevlar-reinforced laminated composites.

Fabric Type	Testing Temperature [°C]	Impact Velocity [m/s]	Absorbed Energy [J]	Conclusion	Remarks	Ref.
Sub-ballistic impact						
Plain weave Kevlar 129	200	400.00	~12.00	This thermal treatment leads to a decrease of 34% in impact energy absorption, compared to untreated woven fabric.	Annealed at 200 °C for 8 h under Ar/H2 gas flow.	[55]
Stacking sequence: [0°/0°/0°/0°/0°/0°/0°/0°]; Matrix type: Epoxy; Impact method: FEM analysis						
Plain weave Kevlar 129	350	400.00	~6.00	This thermal treatment leads to a decrease of 67% in impact energy absorption, compared to untreated woven fabric.	Annealed at 350 °C for 8 h under an Ar/H2 gas flow.	[55]
Stacking sequence: [0°/0°/0°/0°/0°/0°/0°/0°]; Matrix type: Epoxy; Impact method: FEM analysis						
Low-velocity impact						
Neat Kevlar woven fabric	−30	6.00	~8.00	As the temperature increased, the impact strength decreased by 37.5%.	-	[59]
Neat Kevlar woven fabric	60	6.00	~5.00		-	
STF-Kevlar woven fabric	−30	6.00	~17.50	As the temperature increased, the impact strength increased by 85.71%.	-	[59]
STF-Kevlar woven fabric	60	6.00	~32.50		-	
Impact method: Drop hammer impactor						

**Table 6:** Effect of nanoparticles and UV radiation exposure on impact performance of Kevlar fabrics [60].

Fabric Type	UV Irradiation Time [h]	Impact Energy [J]	Absorbed Energy [J]	Remarks
Neat Kevlar	0	24.00	~8.00	-
	80		~6.00	-
	168		~5.00	-
Kevlar with STF, SiO_2_, TiO_2_	0	24.00	~18.00	30% SiO_2_ and 4 wt.% TiO_2_
	80		~16.00	
	168		~14.00	
Kevlar with STF, SiO_2_, TiO_2_	0	24.00	20.23	30% SiO_2_ and 16 wt.% TiO_2_
	80		~18.00	
	168		~16.00	
Impact method: drop weight test equipment with knife blade				

**Table 7:** Densities and tensile properties for different types of glass fibers.

Fiber Type	Young’s Modulus *E* [GPa]	Tensile Strength *σ_t_* [GPa]	Poisson’s Coefficient ν [-]	Elongation at Break *ε* [%]	Density ρ [g/cm^3^]	Ref.
E-glass	72.30	3.445	0.200	4.80	2.580	[70,71]
	72.40	3.450	0.200	4.80	2.540–2.600	[74]
	73.50	3.500	-	4.80	2.570	[73]
	76.00	3.445	-	2.75	2.560	[75]
	83.00–86.00	3.1–3.8	-	4.70	2.540	[76]
S-glass	86.90	4.890	0.220	5.70	2.460	[70,71]
	86.80	4.600	-	5.40	2.460	[73]
	88.00–91.00	4.38–4.59	-	5.40–5.80	2.480–2.490	[77]
C-glass	68.90	3.310	0.276	4.80	2.520	[70,71,74]
D-glass	51.7	2.415	-	4.60	2.110–2.140	[70,71,74]
AR-glass	73.1	3.241	-	4.40	2.700	[70,71,74]
	175.0	3.500	-	2.00	2.680	[73]
	72.0	1.700	-	-	0.845	[78]
R-glass	85.5	4.135	-	4.80	2.540	[70,71,74]

**Table 8:** Influence of fiber type, matrix type, thickness and stacking sequence on the impact behavior of glass fiber reinforced composites and their hybridized configurations.

Fiber Type	Matrix	Thickness [mm]	Stacking Sequence	Impact		Absorbed Energy [J]	Ref.
				E [J]	v [m/s]		
(i) Nonhybridized glass fiber reinforced composites							
Low-velocity impact							
Woven glass	EP *	4.90	[90°/0°/45°/−45°/90°/0°/45°/−45°/90°/0°/45°/−45°]_s_	20.00	2.06	9.01	[82]
	Ph *	5.00	[90°/0°/45°/−45°/90°/0°/45°/−45°/90°/0°/45°/−45°]_s_	20.00	2.06	13.20	
	P *	4.70	[90°/0°/45°/−45°/90°/0°/45°/−45°/90°/0°/45°/−45°]_s_	20.00	2.06	9.49	
	EP *	7.40	[45°/−45°/90°/0°/45°/−45°/90°/0°/45°/−45°/90°/0°/45°/−45°/90°/0°/45°/−45°]	20.00	2.06	11.08	
Impact method: Drop tower system							
Plain woven E-glass	EP *	5.00	[0°]_20_	50.00	-	41.60	[87]
	EP *	5.00	[0°]_20_	150.00	-	125.00	
	EP *	10.00	[0°]_40_	50.00	-	41.20	
	EP *	10.00	[0°]_40_	150.00	-	124.00	
Impact method: FEM analysis							
Unidirectional glass fiber	EP *	4.80	[0°/0°/−45°/+45°/90°/90°]_s_—flat	140.00	5.00	~73.00	[83]
	EP *	4.80	[0°/0°/−45°/+45°/90°/90°]_s_— 304 mm curvature	140.00	5.00	~65.00	
	EP *	4.80	[0°/0°/−45°/+45°/90°/90°]_s_— 760 mm curvature	140.00	5.00	~72.00	
	EP *	4.80	[90°/90°/−45°/+45°/0°/0°]_s_—flat	140.00	5.00	~78.00	
	EP *	4.80	[90°/90°/−45°/+45°/0°/0°]_s_— 304 mm curvature	140.00	5.00	~70.00	
	EP *	4.80	[90°/90°/−45°/+45°/0°/0°]_s_— 760 mm curvature	140.00	5.00	~95.00	
Impact method: Drop tower system							
Unidirectional + glass fiber mats	R *	5.00	[45°/0°/45°]	-	-	293.66	[84]
	R *	6.00	[45°/−45°/90°/0°/45°]	-	-	134.40	
	R *	7.00	[45°/−45°/0°/90°/0°/90°/0°]	-	-	273.57	
	R *	6.00	[0°/45°/0°/−45°/0°/−45°/0°/45°/0]	-	-	458.71	
	R *	8.00	[45°/−45°/0°/0°/0°/0°/0°/0°/−45°/45°]	-	-	191.54	
(ii) Hybridized glass fiber reinforced composites							
Twill woven glass fiber	EP *	5.80	[+45°G/−45 °C/0°G/90 °C]_4s_	33.00	4.34	21.65	[86]
	EP *	5.80	[+45°G/−45°G/0°G/90°G/+45 °C/−45 °C/0 °C/90 °C]_2s_	33.00	4.34	17.93	
Impact method: Drop tower system							

* EP = epoxy, Ph = phenolic, P = polyester, R = resin.

**Table 9:** Effects of testing temperature on the impact behavior of the composite based on PEEK reinforced both with carbon fibers and glass fibers [92].

Testing Temperature: RT [°C]		Testing Temperature: 150 [°C]	
Impact Energy [J]	Absorbed Energy [J]	Impact Energy [J]	Absorbed Energy [J]
25.00	~11.00	25.00	~12.50
30.00	~16.00	30.00	~16.00
35.00	~25.00	35.00	~25.00
40.00	~30.00	40.00	~30.00
Matrix type: Polyether Ether Ketone (PEEK)			
Stacking sequence: [(0°/90°)_G_*/(0°/90°)_C_*/(±45°)_C_*/(0°/90°)_C_*/(±45°)_C_*/(0°/90°)_C_*/(±45°)_C_*/(0°/90°)_C_*]_S_			
Impact method: Charpy pendulum test equipment			

* Subscript G means glass fiber fabric, while subscript C represents satin weave carbon fiber fabric.

**Table 10:** Influence of aging time and temperature on GFRP [95].

		Force [N]		Displacement [mm]	
Aging Time [Weeks]		1	6	1	6
Impact energy [J]	10.00	1679.00	1430.00	7.10	8.37
Temperature [°C]	25.00				
Impact energy [J]	9.00	1411.00	1250.00	8.71	9.79
Temperature [°C]	60.00				
Impact energy [J]	17.00	1950.00	1663.00	11.97	13.06
Temperature [°C]	25.00				
Impact energy [J]	17.00	1815.00	1350.00	13.18	17.00
Temperature [°C]	60.00				
Stacking sequence: [0°/90°]_6_, thickness: 4 mm; matrix type: vinyl ester					
Impact method: Drop weight test system					

**Table 11:** Absorbed energy depending on the impactor geometry acting on flat and curved glass fiber reinforced composite panels [101].

Composite Configuration	Initial Energy [J]	Absorbed Energy [J]	Remarks
Flat panel	18.00	18.00	Spherical impactor
		14.10	Conical impactor
Curved panel: 203 mm ROC *	32.00	31.30	Spherical impactor
		16.90	Conical impactor
Curved panel: 112 mm ROC *	32.00	32.00	Spherical impactor
	32.80	19.80	Conical impactor

* ROC = Radius of Curvature.

**Table 12:** Mechanical properties of the flax, hemp, and jute fibers.

Fiber Diameter [μm]	Elastic Modulus [GPa]	Tensile Strength [MPa]	Strain at Max. Force [%]	Fiber Type	Ref.
Flax fibers					
40.00–600.00	27.60	345.00–1000.00	2.70–3.20	-	[122]
-	50.67	598.73	~1.15	Technical	[104]
19.93	9.23	335.00	0.07 *	Elementary	[123]
82.00	19.40	347.00	1.80	Technical	[124]
Hemp fibers					
25.00–500.00	70.00	690.00	1.60	-	[122]
-	34.65	566.45	~1.10	Technical	[104]
17.00–23.00	30.00–60.00	310.00–750.00	2.00–4.00	-	[121]
123.73	59.05	640.65	1.08 *	Elementary	[125]
Jute fibers					
25.00–200.00	13.00–27.00	393.00–800.00	0.70–1.50	-	[118,122]
5.00–25.00	20.00–55.00	187.00–773.00	1.50–3.10	-	[118]
18.00	13.60	374.00	1.64	-	[115]

* The unit for this strain value is [mm/mm], as stated in the original article [121].

**Table 13:** Influence of hygrothermal aging on the impact performance of flax, hemp, and jute fibers reinforced composite materials.

Composite Configuration	Water Type	Immersion Time	No. of Laminas	Impact Energy [J]	Impact Characteristic BWI		Impact Characteristic AWI		Ref.
					Value	Unit	Value	Unit	
Flax reinforced composites									
35% Flax, 65% EP **	Salt water, RT **	24 h	4	-	270.00	J/m	220.00	J/m	[127]
45% Flax, 55% EP **	Salt water, RT **	24 h	4	-	330.00	J/m	290.00	J/m	
Impact method: Izod test									
Flax, EP **	Deionized water, 23 °C	16 weeks	24	1.00	~2000.00	N	-	-	[129]
Flax, EP **				3.00	~3000.00	N	-	-	
Flax, EP **				5.00	~3300.00	N	-	-	
Flax, EP **				6.00	3450.00	N	3140.00	N	
Impact method: Drop weight test system									
Hemp reinforced composites									
Hemp, polyester AropolTMM604 TBR	Water with salinity 7.8‰	3 months	7	50.00	18.50	J/cm^2^	22.60	J/cm^2^	[130,131]
			9		19.90	J/cm^2^	31.60	J/cm^2^	
			11		21.10	J/cm^2^	35.40	J/cm^2^	
			13		26.80	J/cm^2^	34.70	J/cm^2^	
Impact method: Charpy pendulum test equipment									
Jute reinforced composites									
30% Jute, 70% PP **	-	-	-	-	-		19.02	KJ/m^2^	[133]
Impact method: Charpy pendulum test equipment									
10% Jute, 10% Al_2_O_3_, 80% EP **	Distilled water	10 days	-	-	1.25	J	1.15	J	[132]
30% Jute, 10% Al_2_O_3_, 60% EP **					1.90	J	1.75	J	
40% Jute, 10% Al_2_O_3_, 50% EP **					1.80	J	1.70	J	
Impact method: Izod test									

BWI is the abbreviation for Before Water Immersion, and AWI is the abbreviation for After Water Immersion. ** PP = Polypropylene, EP = Epoxy. RT = Room Temperature.

**Table 14:** Influence of fiber type, matrix type, thickness, and stacking sequence on the impact performance of vegetable fiber reinforced composites.

Composite Configuration	Thickness [mm]	Stacking Sequence	Energy		Impact Strength [kJ/m^2^]	Ref.
			Impacted [J]	Absorbed [J]		
Flax reinforced composites						
Flax *, EP **	4.25	[0°/0°/0°/0°/0°/0°/0°/0°]	3.00	2.00	-	[134]
Flax *, EP **	4.36	[0°/90°/0°/90°/90°/0°/90°/0°]	3.00	2.20		
Flax *, EP **	4.25	[0°/0°/0°/0°/0°/0°/0°/0°]	11.00	10.00		
Flax *, EP **	4.36	[0°/90°/0°/90°/90°/0°/90°/0°]	11.00	9.80		
Flax, VE **	-	[+45°/−45°/+45°/−45°/+45°/−45°/+45°]	50.00	44.76	-	[135]
Impact method: Drop weight test system						
Flax, EP **	-	[0°]–no. of laminas is not specified	-	~4.00	-	[137]
Flax, EP **	-	[15°]–no. of laminas is not specified	-	~2.00		
Flax, EP **	-	[30°]–no. of laminas is not specified	-	~3.00		
Flax, EP **	-	[45°]–no. of laminas is not specified	-	~1.00		
Impact method: Izod test						
Hemp reinforced composites						
Hemp, EP **	-	[0°]–no. of laminas is not specified	-	~5.00	-	[137]
Hemp, EP **	-	[15°]–no. of laminas is not specified	-	~3.20		
Hemp, EP **	-	[30°]–no. of laminas is not specified	-	~4.00		
Hemp, EP **	-	[45°]–no. of laminas is not specified	-	~2.20		
Impact method: Izod test						
Hemp, EP**	-	[0°/0°/0°]	-	~2.50	-	[138]
Jute reinforced composites						
Jute, EP **	-	[0°/0°/0°]	-	~6.00	-	[138]
Jute, HDPE *	6.70	[0°/0°/0°]	-	-	21.10	[139]
Impact method: Izod test						

* Flax fiber corresponding to the results presented in this table is normal, not fibrillated. ** EP = Epoxy, VE = Vinyl Ester, HDPE = High-Density Polyethylene.

**Table 15:** Impact performance of polymer composites reinforced with Kevlar fibers hybridized with vegetable fibers.

Vegetable Fiber *	Matrix	Nanofiller ** [wt.%]	Impact Energy [J]	Impact Velocity [m/s]	Absorbed Energy [J]	Ref.
Low-velocity impact						
[K/H/K/H/K]	Epoxy 65.0 wt.%	CP 2.5	-	6.00	3.52	[148]
	Epoxy 62.5 wt.%	CP 5	-		3.65	
	Epoxy 60.0 wt.%	CP 7.5	-		2.70	
Impact method: Charpy pendulum test equipment						
[K/F/H/H/F/K]	Epoxy	-	6.00	-	4.58	[147]
		-	12.00	-	5.36	
		-	18.00	-	9.20	
		-	24.00	-	8.60	
Impact method: Drop weight test system						
[K0°/J90°/K0°/J90°/K0°/J90°/K0°]	Epoxy	-	-	3.50	75.66	[149]
[K0°/J45°/K0°/J45°/K0°/J45°/K0°]		-	-	3.50	68.52	
Impact method: Charpy pendulum test equipment						
Sub-ballistic impact						
[CK/F/F/F/F]_s_	Bio-phenolic/ Epoxy	-	274.82	331.55	137.35	[144]
[CK/CK/F/F/F/F/ F/CK/CK]		-	310.69	352.52	139.63	
[CK/CK/CK/F/F/F/ CK/CK/CK]		-	294.51	343.22	142.55	
Impact method: single-stage gun setup						

* K = Kevlar; H = Hemp; F = Flax; J = Jute; CK = Carbon and Kevlar fabric. ** CP = Coconut and Palm powder.

**Table 16:** Impact performance of polymer composites reinforced with glass fibers hybridized with vegetable fibers.

Vegetable Fiber *	Matrix *	Impact Energy [J]	Impact Velocity [m/s]	Energy Peak [J]	Impact Strength [kJ/m^2^]	Ref.
Glass-jute hybrid composites						
J20 wt.%, G0 wt.%	PP	5.00	-	-	25.41	[150]
J15 wt.%, G5 wt.%	PP	5.00	-	-	38.02	
J10 wt.%, G10 wt.%	PP	5.00	-	-	42.79	
J5 wt.%, G15 wt.%	PP	5.00	-	-	53.34	
Impact method: Charpy pendulum test equipment						
[J/J/G/J/J]	PBS	-	5.50	-	43.00	[151]
[J/G/J/G/J]	PBS	-	5.50	-	54.00	
[G/J/J/J/G]	PBS	-	5.50	-	57.00	
[G/J/G/J/G]	PBS	-	5.50	-	65.00	
[J/G/G/G/J]	PBS	-	5.50	-	63.00	
[G/G/J/G/G]	PBS	-	5.50	-	70.50	
Impact method: Izod test						
Core Glass fiber wrapped by jute fiber	EP	-	3.46	-	65.35	[155]
GO-treated core glass fiber wrapped by jute fiber	EP	-	3.46	-	85.56	
Impact method: Izod test						
[J0°/G90°/J90°/G0°]	EP	5.00	-	-	25.99	[156]
KOH treated [J0°/G90°/J90°/G0°]	EP	5.00	-	-	27.80	
Impact method: Charpy pendulum test equipment						
Glass-flax hybrid composites						
[F/F/F/G/F/F/F/F]	EP	50.00	4.25	3.558	-	[157]
[F/F/F/G/G/F/F/F]	EP	50.00	4.25	4.322	-	
[F/G/F/F/F/F/G/F]	EP	50.00	4.25	7.103	-	
[F/F/G/G/G/G/F/F]	EP	50.00	4.25	9.024	-	
Impact method: Drop weight test system						

* J = Jute; F = Flax; G = Glass; C = Carbon; GO = Graphene Oxide; EP = Epoxy; PP = Polypropylene; PBS = Polybutylene Succinate.

## Data Availability

No new data were created or analyzed in this study. Data sharing is not applicable to this article.
